# Diverse GABAergic neurons organize into subtype‐specific sublaminae in the ventral lateral geniculate nucleus

**DOI:** 10.1111/jnc.15101

**Published:** 2020-06-24

**Authors:** Ubadah Sabbagh, Gubbi Govindaiah, Rachana D. Somaiya, Ryan V. Ha, Jessica C. Wei, William Guido, Michael A. Fox

**Affiliations:** ^1^ Center for Neurobiology Research Fralin Biomedical Research Institute at Virginia Tech Carilion Roanoke VA USA; ^2^ Graduate Program in Translational Biology, Medicine, and Health Virginia Tech Blacksburg VA USA; ^3^ Department of Anatomical Sciences and Neurobiology University of Louisville School of Medicine Louisville KY USA; ^4^ School of Neuroscience Virginia Tech Blacksburg VA USA; ^5^ NeuroSURF Fralin Biomedical Research Institute at Virginia Tech Carilion Roanoke VA USA; ^6^ Department of Biological Sciences Virginia Tech Blacksburg VA USA; ^7^ Department of Pediatrics Virginia Tech Carilion School of Medicine Roanoke VA USA

**Keywords:** circuit, GABAergic, geniculate, retina, thalamus, visual

## Abstract

In the visual system, retinal axons convey visual information from the outside world to dozens of distinct retinorecipient brain regions and organize that information at several levels, including either at the level of retinal afferents, cytoarchitecture of intrinsic retinorecipient neurons, or a combination of the two. Two major retinorecipient nuclei which are densely innervated by retinal axons are the dorsal lateral geniculate nucleus, which is important for classical image‐forming vision, and ventral LGN (vLGN), which is associated with non‐image‐forming vision. The neurochemistry, cytoarchitecture, and retinothalamic connectivity in vLGN remain unresolved, raising fundamental questions of how it receives and processes visual information. To shed light on these important questions, used in situ hybridization, immunohistochemistry, and genetic reporter lines to identify and characterize novel neuronal cell types in mouse vLGN. Not only were a high percentage of these cells GABAergic, we discovered transcriptomically distinct GABAergic cell types reside in the two major laminae of vLGN, the retinorecipient, external vLGN (vLGNe) and the non‐retinorecipient, internal vLGN (vLGNi). Furthermore, within vLGNe, we identified transcriptionally distinct subtypes of GABAergic cells that are distributed into four adjacent sublaminae. Using trans‐synaptic viral tracing and in vitro electrophysiology, we found cells in each these vLGNe sublaminae receive monosynaptic inputs from retina. These results not only identify novel subtypes of GABAergic cells in vLGN, they suggest the subtype‐specific laminar distribution of retinorecipient cells in vLGNe may be important for receiving, processing, and transmitting light‐derived signals in parallel channels of the subcortical visual system.

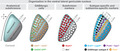

## Graphical abstract

The ventral lateral geniculate nucleus (vLGN) is part of the visual thalamus. It can broadly be separated into two structural domains or laminae, the external vLGNe (which receives retinal input) and the internal vLGNi (receives no retinal input). In this study, we describe subtypes of transcriptomically distinct GABAergic neurons that populate the vLGN and organize into discrete, adjacent sublaminae in the vLGNe. Taken together, our results show four subtype‐specific sublaminae of retinorecipient neurons in vLGNe.

**Abbreviations**
AAV: adeno‐associated virus
CTB: cholera toxin subunit b
DAPI: 4′,6‐diamidino‐2‐phenylindole
dLGN: dorsal lateral geniculate nucleus
EPSC: excitatory post‐synaptic current
GABA: gamma aminobutyric acid
GFP: green fluorescent protein
IGL: intergeniculate leaflet
IHC: immunohistochemistry
ISH: in situ hybridization
LGN: lateral geniculate nucleus
OT: optic tract
PPD: paired pulse depression
RGC: retinal ganglion cells
RRID: research resource identifier
SC: superior colliculus
SCN: suprachiasmatic nucleus
tdt: tdTomato
vLGN: ventral lateral geniculate nucleus
vLGNe: external vLGN
vLGNi: internal vLGN
YFP: yellow fluorescent protein

## 1 INTRODUCTION

Information about the visual world is captured by the retina and transmitted by retinal ganglion cells (RGCs) to a diverse array of retinorecipient nuclei, including those in thalamic, hypothalamic, and midbrain regions (Fleming, Benca, & Behan, 2006; Gaillard, Karten, & Sauvé, 2013; Martersteck et al., 2017; Monavarfeshani, Sabbagh, & Fox, 2017; Morin & Studholme, 2014). There is an organizational logic to these long‐range retinal projections where RGCs, of which there are more than three dozen morphologically and functionally distinct subtypes, project to distinct and sometimes mutually exclusive retinorecipient regions (Berson, 2008; Dhande et al., 2011; Dhande, Stafford, Lim, & Huberman, 2015; Hattar et al., 2006; Kay et al., 2011; Osterhout et al., 2011; Yonehara et al., 2009). Many of these retinorecipient nuclei are critical to the execution of specific visual behaviors. For instance, retinal inputs to the dorsal lateral geniculate nucleus (dLGN) are important for image‐formation and direction selectivity, those to the superior colliculus (SC) are important for gaze control, those to pretectal nuclei are important for pupillary reflexes and image stabilization, and those to the suprachiasmatic nucleus are important for circadian photoentrainment (Dhande et al., 2015; Piscopo, El‐Danaf, Huberman, & Niell, 2013; Seabrook, Burbridge, Crair, & Huberman, 2017). Not only do RGCs project to different retinorecipient nuclei, but projections of distinct RGC subtypes are also segregated within a single retinorecipient region. For example, it has long been appreciated that projections from transcriptomically distinct ipsilateral and contralateral RGCs terminate in distinct domains of most rodent retinorecipient nuclei (Godement, Salaün, & Imbert, 1984; Jaubert‐Miazza et al., 2005; Morin & Studholme, 2014; Muscat, Huberman, Jordan, & Morin, 2003; Wang, Marcucci, Cerullo, & Mason, 2016)

A long‐standing objective of visual neuroscientists has been to characterize cell‐type‐specific circuits in these retinorecipient regions, in terms of both inputs from RGCs and outputs to distinct downstream brain regions. For example, that distinct subtypes of RGCs terminate in different sublaminae of the SC (Dhande & Huberman, 2014; Huberman et al., 2008, 2009; Kim, Zhang, Meister, & Sanes, 2010; Martersteck et al., 2017; Oliveira & Yonehara, 2018). Post‐synaptic to these retinal inputs are at least four morphologically and functionally distinct classes of retinorecipient neurons which are stellate, horizontal, wide‐field, and narrow‐field cells (Gale & Murphy, 2014, 2016). Identifying subtype‐specific retinocollicular circuitry facilitated the discovery that specific collicular cell types participate in different aspects of visually guided behavior (Hoy, Bishop, & Niell, 2019; Reinhard et al., 2019; Shang et al., 2015, 2018).

The dLGN, which processes and relays classical image‐forming visual information to primary visual cortex, shares an organizational feature with SC in the kinds of retinal afferents it receives, where subtype‐specific arborization of RGC axons been clearly characterized and forms so‐called “hidden laminae” (Hong & Chen, 2011; Martin, 1986; Reese, 1988). These hidden layers have been revealed by methods which individually label functionally and morphologically distinct classes of RGCs using transgenic reporter mouse lines (Huberman et al., 2008, 2009; Kay et al., 2011; Kim et al., 2010; Kim, Zhang, Yamagata, Meister, & Sanes, 2008). The dLGN is populated by just a few types of retinorecipient neurons, which include three classes of thalamocortical relay cells (X‐like, Y‐like, and W‐like) and 1–2 classes of GABAergic interneurons (Arcelli, Frassoni, Regondi, Biasi, & Spreafico, 1997; Jaubert‐Miazza et al., 2005; Krahe, El‐Danaf, Dilger, Henderson, & Guido, 2011; Leist et al., 2016; Ling, Hendrickson, & Kalil, 2012). While their organization is not as ordered as their retinal afferents, classes of dLGN relay cells exhibit some regional preferences in their distribution, whereas interneurons are evenly dispersed throughout the nucleus (Krahe et al., 2011). Cell type‐specific circuitry and function has also been demonstrated in dLGN, where W‐like relay neurons receive input from direction‐selective RGCs and in turn project to the superficial layers of mouse primary visual cortex (Cruz‐Martín et al., 2014).

While our understanding of subtype‐specific circuits has facilitated functional studies of SC and dLGN, there remain many retinorecipient regions about which such foundational information is unknown. One such region is the ventral LGN (vLGN), a portion of ventral thalamus that neighbors dLGN and is similarly innervated by retinal axons. Although less studied, it has been shown that vLGN is remarkably distinct from its dorsal counterpart in its transcriptome, proteome, cytoarchitecture, and circuitry (Harrington, 1997; Monavarfeshani et al., 2018; Sabbagh et al., 2018; Su et al., 2011). In fact, distinct subtypes of RGCs project to vLGN and dLGN, and the majority of dLGN‐projecting RGC classes fail to send collateral axons into vLGN, despite having to pass by it (or through it) on the way to dLGN (Huberman et al., 2008, 2009; Kim et al., 2008). Retinal axons that target vLGN terminate in a lateral subdivision known as the external vLGN (vLGNe), which is cytoarchitectonically distinct from the internal vLGN (vLGNi) which receives little, if any, retinal input (Gabbott & Bacon, 1994; Harrington, 1997; Niimi, Kanaseki, & Takimoto, 1963; Sabbagh et al., 2018). The identity of retinorecipient cells in vLGNe remains largely unknown, although it is likely to include GABAergic cells (Huang et al., 2019), which represent the prevalent type of neuron in vLGN (Gabbott & Bacon, 1994; Harrington, 1997; Inamura, Ono, Takebayashi, Zalc, & Ikenaka, 2011).

Here, to address these gaps, we sought to determine the cell‐types populating the vLGN, and their connectivity to retinal afferents. We assessed vLGN neurochemistry and cytoarchitecture by labeling cells with canonical and novel cell type markers. We found a richly diverse and tightly organized cellular landscape in vLGN, where transcriptomically distinct cell types are distributed in laminar subdomains, which appear to receive monosynaptic inputs from the retina. Our findings not only identify a novel organization of retinorecipient cells in vLGN, they suggest this order may be important for receiving, processing, and transmitting distinct light‐derived signals in parallel channels of the subcortical visual system.

## 2 MATERIALS AND METHODS

### 2.1 Animals

Wild‐type C57BL/6 mice were obtained from Jackson Laboratory. We obtained the following mice from Jackson Laboratory: *Pvalb‐Cre* (JAX #: 008069, RRID:IMSR_JAX:008069), *Gad2‐Cre* (JAX #: 010802, RRID:IMSR_JAX:010802), *Sst‐Cre* (JAX #: 028864, RRID:IMSR_JAX:028864), *Sun1‐stop‐GFP* (JAX #: 021039, RRID:IMSR_JAX:021039), *Thy1‐STOP‐YFP* (JAX #: 005630, RRID: IMSR_JAX:005630), *ROSA‐stop‐tdT* (JAX #: 007909, RRID:IMSR_JAX:007909), and *GAD67‐GFP* (JAX #: 007673, RRID:IMSR_JAX:007673). Animals were housed in a temperature‐controlled environment, in a 12 hr dark/light cycle, and with access to food and water ad libitum. Both males and females were used in these experiments. Genomic DNA was isolated from tails genotyping as previously described (Su, Gorse, Ramirez, & Fox, 2010) using the following primers: *yfp*: fwd‐AAGTTCATCTGCACCACCG, rev‐TCCTTGAAGAAGATGGTGCG; *cre*: fwd‐TGCATGATCTCCGGTATTGA, rev‐CGTACTGACGGTGGGAGAAT; *sun1*: fwd‐CTTCCCTCGTGATCTGCAAC, mut_rev‐ GTTATGTAACGCGGAACTCCA, wt‐rev: CAGGACAACGCCCACACA; *tdt*: fwd‐ ACCTGGTGGAGTTCAAGACCATCT, rev‐TTGATGACGGCCATGTTGTTGTCC. Animals were maintained and experiments conducted in compliance with National Institutes of Health (NIH) guidelines and approved protocols of the Virginia Polytechnic Institute and State University Institutional Animal Care and Use Committee (IACUC# 18‐165 and 18‐142). Unless otherwise stated, *n* = number of animals and age‐matched wild‐type or transgenic reporters were used where multiple animals were compared. This study was not preregistered, and no blinding or randomization was performed. In total, 38 mice were used in these studies. No animals used in the experiments described were excluded from analyses performed here.

### 2.2 Immunohistochemistry (IHC)

Mice were intraperitoneally injected with a lethal dose of freshly prepared 12.5 μg/mL tribromoethanol (Avertin) followed by transcardial perfusion with PBS and 4% paraformaldehyde (PFA; pH 7.4) as previously described (Sabbagh et al., 2018) and as approved by the Virginia Polytechnic Institute and State University IACUC. Tribromoethanol was selected since it has minimal impact on the cardiovascular system and was prepared by dissolving 2.5 g of 2,2,2 tribromoethanol in 5 mL of 2‐methyl‐2‐butanol, adding 200 mL ddH_2_O (7.4 pH), then filter sterilizing the solution through a 0.5 μm filter. Extracted brains were kept in 4% PFA overnight at 4°C, and then incubated for at least 48 hr in 30% sucrose in PBS. Fixed tissues were embedded in Tissue Freezing Medium (Electron Microscopy Sciences) and cryosectioned at 30 μm sections on a Leica CM1850 cryostat. Sections were air‐dried onto Superfrost Plus slides (Fisher Scientific) for 15 min before being incubated in blocking buffer (2.5% bovine serum albumin, 5% Normal Goat Serum, 0.1% Triton‐X in PBS) for 1 hr at room temperature (or at 22°C). Primary antibodies were diluted in blocking buffer at the following dilutions and incubated on tissue sections at 4°C overnight: Calb1 (Swant, CB‐38a, RRID:AB_10000340, 1:1,000) and Pvalb (Millipore‐Sigma, MAB1572, RRID:AB_2174013, 1:1,000). Sections were then washed three times PBS and incubated in anti‐mouse or anti‐rabbit fluorescently conjugated secondary antibodies (Invitrogen Life Technologies, RRID: AB_2535805 and AB_2633282) diluted in blocking buffer (1:1,000) for 1 hr at 22°C. Tissue sections were then washed at least three times with PBS, stained with 4′,6‐diamidino‐2‐phenylindole (DAPI) (1:5,000 in water), and mounted using Vectashield (Vector Laboratories, RRID:AB_2336789).

### 2.3 Riboprobe production

Riboprobes were generated as previously described (Monavarfeshani et al., 2018; Su et al., 2010). *Gad1* (Gad1‐F: TGTGCCCAAACTGGTCCT; Gad1‐R: TGGCCGATGATTCTGGTT; NM_001312900.1; nucleotides 1099–2081), *Lypd1* (Lypd1‐F: AAGGGAGTCTTTTTGTTCCCTC; Lypd1‐R: TACAACGTGTCCTCTCAGCAGT; NM_145100.4; nucleotides 849–1522), *Arx* (Arx‐F: CTGAGGCTCAAGGCTAAGGAG; Arx‐R: GGTTTCCGAAGCCTCTACAGTT; NM_007492.4; nucleotides 1830–2729), *Ecel1* (Ecel1‐F: CGCGCTCTTCTCGCTTAC; Ecel1‐R: GGAGGAGCCACGAGGATT; NM_001277925.1; nucleotides 942–1895), *Nxph1* (Nxph1‐F: ATAGGACAGGGCTGTCACCTTA; Nxph1‐R: TTACTGAGAACAAGCTCCTCCC; NM_008751.5; nucleotides 1095–1695), *Spp1* (Spp1‐F: AATCTCCTTGCGCCACAG; Spp1‐R: TGGCCGTTTGCATTTCTT; NM_001204201.1; nucleotides 309–1263), *Sst* (NM_009215.1; nucleotides 7–550), and *Penk* (Penk‐F: TTCCTGAGGCTTTGCACC; Penk‐R: TCACTGCTGGAAAAGGGC; NM_001002927.3; nucleotides 312–1111) cDNAs were generated using Superscript II Reverse Transcriptase First Strand cDNA Synthesis kit (#18064014, Invitrogen) according to the manufacturer manual, amplified by PCR using primers designed for the above gene fragments, gel purified, and then cloned into a pGEM‐T Easy Vector using pGEM‐T Easy Vector kit, (#A1360, Promega) according to the kit manual. Anti‐sense riboprobes against target genes were synthesized from 5 µg linearized plasmids using digoxigenin‐(DIG) or fluorescein‐labeled uridylyltransferase (UTP) (#11685619910, #11277073910, Roche, Mannheim, Germany) and the MAXIscript in vitro Transcription Kit (#AM1312, Ambion) according to the kit manual. Five microgram of riboprobe (20 µl) was hydrolyzed into ~0.5 kb fragments by adding 80 µl of water, 4 µl of NaHCO_3_ (1 M), 6 µl Na_2_CO_3_ (1 M), and incubating the mixture in 60°C. RNA fragments were finally precipitated in ethanol and resuspended in RNAase‐free water.

### 2.4 In situ hybridization (ISH)

ISH was performed on 30‐μm‐thin cryosections as described previously (Sabbagh et al., 2018). Sections were first allowed to air dry for 1hr at room temperature and washed with PBS for 5 min to remove freezing media. They were then fixed in 4% PFA for 10 min, washed with PBS for 15 min, incubated in proteinase K solution for 10 min, washed with PBS for 5 min, incubated in 4% PFA for 5 min, washed with PBS for 15 min, incubated in acetylation solution for 10 min, washed with PBS for 10 min, incubated in PBS‐diluted 0.1% triton for 30 min, washed with PBS for 40 min, incubated in 0.3% H_2_O_2_ for 30 min, washed with PBS for 10 min, pre‐hybridized with hybridization solution for 1 hr, before being hybridized with heat‐denatured riboprobes at 62.5°C overnight. Sections were then washed for five times in 0.2X SSC buffer at 65°C. Slides were then washed with TBS, blocked, and incubated with horseradish peroxidase‐conjugated anti‐DIG (#11207733910, Roche, RRID:AB_514500) or anti‐fluorescein antibodies (#11426346910, Roche, RRID:AB_840257) overnight at 4°C. Lastly, bound riboprobes were detected by a tyramide signal amplification system (#NEL753001KT, PerkinElmer).

### 2.5 Anterograde axon and mono‐synaptic tracing

Intravitreal injection of cholera toxin subunit B (to trace retinal terminals) was performed as previously described (Monavarfeshani et al., 2018; Su et al., 2011). Briefly, mice were anesthetized with isoflurane, and 1 μl of 1 mg/ml fluorescently conjugated Alexa‐647‐CTB (Invitrogen, C34778) was binocularly injected with a fine glass pipette using a picospritzer. The rapid, minor, and non‐invasive nature of these injections (which is not considered a surgery) precluded the need for additional analgesics. After 3 days, animals were killed and transcardially perfused with PBS followed by PFA. We opted for a binocular injection here because it is the best way to accurately delineate the border between vLGNe and vLGNi.

A similar intravitreal injection of AAV2/1‐hSyn‐Cre‐WPRE‐hGH (2.5 × 10^13^ GC/mL, here referred to as AAV1‐Cre) was used to monosynaptically label retinorecipient neurons in the vLGN. 1.2 μl of AAV‐Cre virus was binocularly injected at an approximate 45° angle relative to the optic axis. We opted for a binocular injection here to maximize the probability of AAV‐Cre infecting RGCs and the probability of trans‐synaptic infection in vLGN. AAV1‐Cre was a gift from James M. Wilson (Addgene viral prep #105553‐AAV1; RRID:Addgene_105553). Animals were killed and perfused with PFA as described above 6–10 weeks after injection.

### 2.6 Transcriptomic analyses

RNA from wild‐type vLGN and dLGN was extracted and purified from mice at P3, P8, P12, and P25, then processed at the Genomics Research Laboratory at Virginia Tech's Biocomplexity Institute for RNAseq analysis. The RNA sequencing experiment was previously published and the protocol is described in detail in (Monavarfeshani et al., 2018).

### 2.7 In vitro slice preparation and whole‐cell recording

In vitro recordings were conducted on genetically labeled vLGN neurons using methods described previously (Hammer et al., 2014). Mice were anesthetized with isoflurane, decapitated and brains were rapidly immersed in an ice‐cold, oxygenated (95% O_2_/5% CO_2_) solution containing the following (in mM): 26 NaHCO_3_, 234 sucrose, 10 MgCl_2_, 11 glucose, 2.5 KCl, 1.25 NaH_2_ PO_4_, 2 CaCl_2_. Coronal sections (270 µm) containing dLGN and vLGN were cut on a vibratome and placed in a chamber containing artificial cerebral spinal fluid (ACSF; in mM: 126 NaCl, 2.5 KCl, 1.25 NaH_2_PO_4_, 2.0 MgCl_2_, 26 NaHCO_3_, 2 CaCl_2_, 2 MgCl_2_, and 10 glucose, saturated with 95% O_2_/5% CO_2_, pH 7.3) at 32°C for 30 min and then at room temperature (21°C). Individual slices were transferred to a recording chamber maintained at 32°C and perfused continuously at a rate of 2.5 ml/min with oxygenated ACSF. Borosilicate pipettes were pulled using a two‐step puller (Narishige) and filled with a solution containing the following (in mM): 117 K‐gluconate, 13 KCl,1 MgCl_2_, 0.07 CaCl_2_, 0.01 EGTA, 10 HEPES, 2 Na‐ATP, and 0.4 Na‐GTP (pH 7.3, 290 osmol/L). For all recordings, biocytin (0.5%, Sigma) was included in the internal solution for intracellular filling and 3‐D neuron reconstruction using confocal microscopy (Charalambakis, Govindaiah, Campbell, & Guido, 2019; El‐Danaf et al., 2015; Krahe et al., 2011). The final tip resistance of filled electrodes was 6–8 MΩ.

Whole‐cell patch recordings were made in current and voltage clamp using an amplifier (Multiclamp700B, Molecular Devices), filtered at 3–10 kHz, digitized (Digidata 1440A) at 20kHz and stored on computer. Pipette capacitance, series resistance, input resistance, and whole‐cell capacitance were monitored throughout the recording session devices.

To examine the intrinsic membrane properties of vLGN neurons, the voltages responses triggered by current step injection (−120 to + 200 pA, 20 pA pulses, 600 ms) were recorded at resting membrane levels. Synaptic responses were recorded in voltage clamp (holding potential of −70mV) and evoked by electrical stimulation of the optic tract (OT) using bipolar tungsten electrodes (0.5 MΩ; A‐M Systems) positioned just below the ventral border of vLGN. OT stimulation consisted of a 10Hz (10 pulses) train delivered at an intensity (25–200 µA) that evoked a maximal response (Hammer et al., 2014; Jaubert‐Miazza et al., 2005). In vitro data are obtained from all healthy cells which we were able to record from in six *Pvalb‐Cre::Thy1‐Stop‐YFP* mice, three *Sst‐cre::Rosa‐Stop‐tdT* mice, and two *GAD67‐GFP* mice.

### 2.8 Statistics

Since comparisons of electrophysiological measurements were between three groups and were not all normally distributed (D'Agostino‐Pearson test; all groups were normally distributed except for the GAD67‐GFP group because of sample size; GraphPad Prism), we determined statistical significance by a Kruskal–Wallis test with Dunn's correction for multiple comparisons using GraphPad Prism (version 8.0.; RRID:SCR_002798). Differences were considered significant when *p* < .05. We did not perform outlier tests for electrophysiological data. No exclusion criteria were predetermined before the described experiments were performed and no data (or animals) were excluded from any of the analysis.

### 2.9 Quantification and imaging

No sample calculation was performed; a sample size of at least three biological replicates (mice) was determined to be appropriate (based on observed variability and previous experience (Sabbagh et al., 2018)) where quantitative comparison across replicates was performed (colocalization with *Gad1* signal, spatial distribution analysis in vLGNe/vLGNi using cholera toxin subunit b (CTB) tracing, and line scan analyses). In these analyses, we obtained measurements obtained from three to four vLGN sections per mouse brain and averaged them to obtain the mean value for that biological replicate.

To quantify the GABAergic neurons as a percentage of total cells in vLGN and dLGN, we labeled and counted *Gad1* ^+^ (by ISH), *Gad2* ^+^ (by *Gad2‐Cre::Sun1‐Stop‐GFP* transgenic reporter), and *GAD67* ^+^ *‐GFP* (by *GAD67‐GFP* transgenic reporter) neurons and divided by all cells counted in that section by DAPI counter‐staining. To quantify the density of a given GABAergic subtype in the two main subdomains of vLGN, we labeled with the respective subtype marker (after intravitreal CTB injection) and counted cells in the retinorecipient (CTB^+^) vLGNe and non‐retinorecipient (CTB^−^) vLGNi and normalized to the area of the respective vLGN subdomain. Areas were measured by manual outlining of the border of vLGNe or vLGNi using ImageJ software (version 1.52n, NIH). Boundaries of vLGN or dLGN were determined by DAPI counter‐staining. For these imaging experiments, no prior sample calculation was performed; a sample size of at least 3 biological replicates (mice) was determined to be appropriate based on observed variability and previous experience to asses colocalization with *Gad1* signal, spatial distribution analysis in vLGNe/vLGNi using CTB tracing, and line scan analyses (described below). For each of these analyses, we obtained measurements from three to four vLGN sections per mouse brain and averaged them to obtain the mean value for that biological replicate.

To quantify the spatial distribution of cell‐type marker expression across the entire vLGN, we developed a custom line scan script (khatScan) that runs in ImageJ which overlays the vLGN with equally spaced lines. We opted for this approach over manually drawing lines to avoid user bias. A brief summary of how this script works: to determine the curvature of the vLGN in a particular image, khatScan prompts the user to draw a line along the optic tract adjacent to the vLGN, then automatically draws lines of a set length and number guided by that curve and plots the signal intensity across the x coordinates of each line. These intensities can then be averaged to determine where there is a specific enrichment for that marker in the vLGN. All imaging for quantification was performed on a confocal Zeiss LSM 700 microscope at 20x magnification and 0.5 digital zoom.

## 3 RESULTS

### 3.1 Identification of distinct subtypes of GABAergic cells in vLGN

**FIGURE 1:**
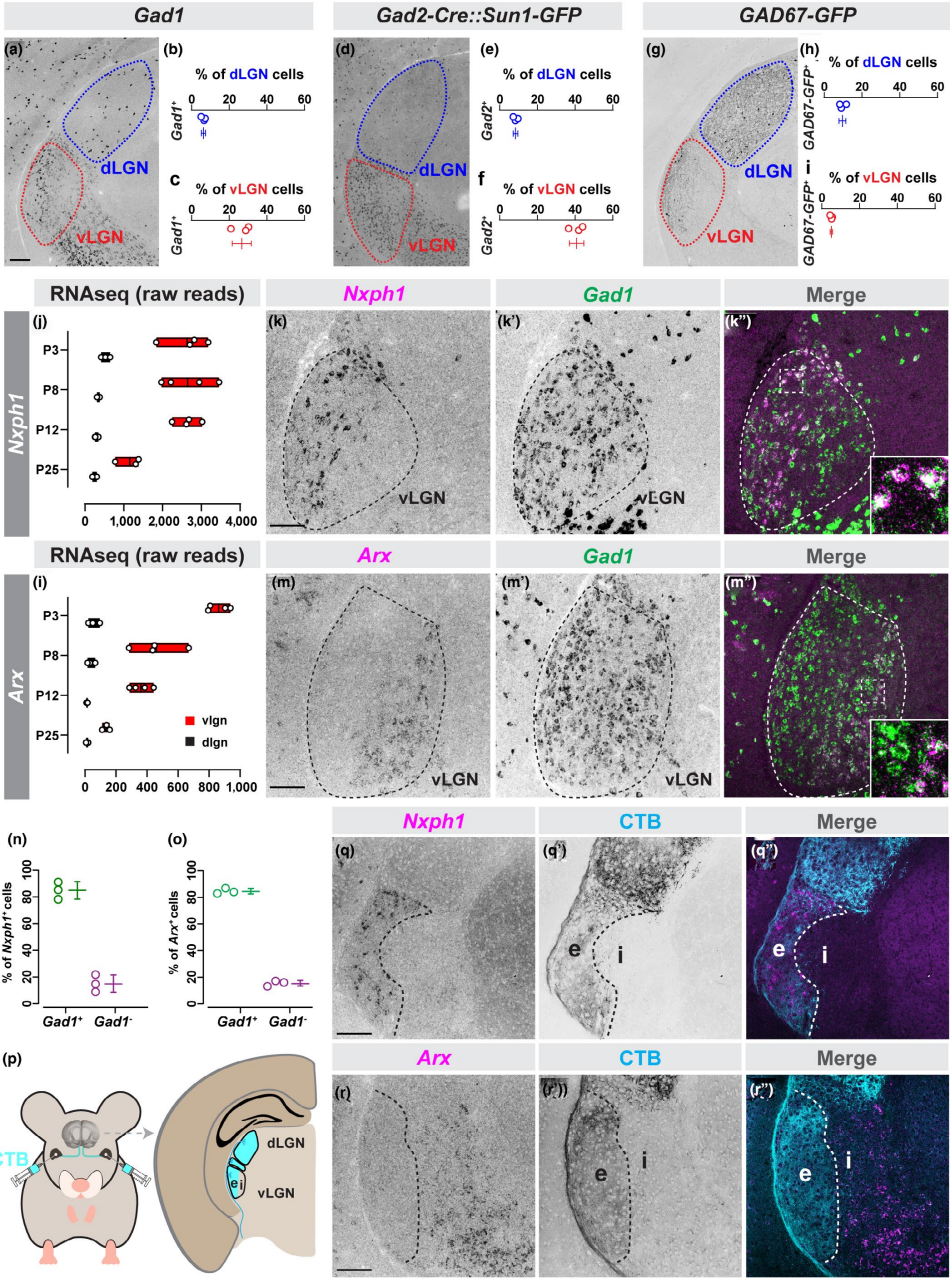
Cells in retinorecipient and non‐retinorecipient zones of vLGN are molecularly distinct. (a) In situ hybridization for Gad1 mRNA in P60 dLGN and vLGN. (b and c) Quantification of the percentage of DAPI^+^ cells that are *Gad1* ^+^ in dLGN (b) and vLGN (c). Data points represent biological replicates, bars represent mean ± *SD*. (d) Transgenic labeling of *Gad2* ^+^ GABAergic neurons in P60 LGN of *Gad2‐Cre*::*Sun1‐Stop‐GFP* mice. (e and f) Quantification of the percentage of DAPI+cells that are *Gad2* ^+^ in dLGN (e) and vLGN (f) of *Gad2‐Cre*::*Sun1‐Stop‐GFP*. Data presented as in (c). (g) Transgenic labeling of GFP^+^ cells in P60 LGN of *GAD67‐GFP* mice. (h and i) Quantification of the percentage of DAPI^+^ cells that are GFP^+^ in dLGN (h) and vLGN (i) of *GAD67‐GFP* mice. Data presented as in (c). (j) Raw transcript reads of *Nxph1* mRNA in vLGN and dLGN obtained by RNAseq. Individual data points plotted as white circles, min/max values are confined to the red bars, and vertical black line with bars depicts mean. (k‐k’’) Double in situ hybridization (ISH) for *Nxph1* and *Gad1* mRNAs in P10 vLGN. (l) Raw transcript reads of *Arx* mRNA in vLGN and dLGN obtained by RNAseq, presented as in (j). (m‐m’’) Double ISH in vLGN using riboprobes generated against *Arx* and *Gad1* mRNAs in P10 vLGN. (n and o) Quantification of the percent of *Nxph1* ^+^ (n) or *Arx* ^+^ (o) cells that co‐express *Gad1* mRNA. Data presented as in (c). (p) Intravitreal injection of Alexa‐conjugated Cholera Toxin subunit B (CTB) labels retinothalamic projections. (q and r) ISH for *Nxph1* (q) and *Arx* (r) mRNAs in P10 CTB‐labeled vLGN. *n* = 3 animals in all data presented. All scale bars = 100 µm

We first determined precisely what proportion of vLGN cells were GABAergic. In the brain, GABAergic neurons express *Gad1* and/or *Gad2*, genes which encode glutamate decarboxylases (GAD67 and GAD65, respectively), the biosynthetic enzymes necessary for the production of the neurotransmitter GABA. We therefore took two complementary approaches to label GABAergic interneurons: we performed in situ hybridization (ISH) with riboprobes generated against *Gad1* mRNA and we crossed *Gad2‐Cre* mice (in which the Cre recombinase is expressed under the control of *Gad2* promoter) to *Rosa‐Stop‐Sun1/sfGFP* mice to transgenically label *Gad2*‐expressing cells with a GFP‐tagged nuclear protein (Mo et al., 2015). Both approaches revealed a dramatic enrichment of GABAergic cells in vLGN compared to dLGN, as expected from previous studies (Figure 1a–i) (Langel, Ikeno, Yan, Nunez, & Smale, 2018; Yuge et al., 2011). We found that > 25% of cells in vLGN were *Gad1* ^+^ (Figure 1c). A slightly higher percentage of vLGN cells (~40%) were GFP^+^ in *Gad2‐Cre::Sun1‐Stop‐GFP* mice (Figure 1f). This increased percentage could be attributable to the limitations of mRNA detection by ISH and therefore represents a more accurate picture of the overall population of GABAergic cells in vLGN. This same approach labeled less than 10% of cells in dLGN, a number in line with previous reports (Arcelli et al., 1997; Evangelio, García‐Amado, & Clascá, 2018; Su et al., 2020).

Several groups, including our own, have previously used a *GAD67‐GFP* transgenic line to label most (if not all) GABAergic cells in dLGN (Charalambakis et al., 2019; Seabrook, Krahe, Govindaiah, & Guido, 2013; Su et al., 2020). However, few GABAergic neurons are labeled in vLGN of these mice (Figure 1g). In fact, we found less than 5% of cells in vLGN were GFP^+^ in *GAD67‐GFP* (Figure 1i). The dramatically fewer GFP^+^ cells, compared to the number of GABAergic cells observed by labeling with either *Gad1* ISH or *Gad2‐Cre::Sun1‐Stop‐GFP,* is because of the fact that the *GAD67‐GFP* labels only a subset of thalamic GABAergic cells—likely local inhibitory interneurons—in visual thalamus (Su et al., 2020).

Together, these results suggested that multiple subtypes of GABAergic cells exist in vLGN, unlike in dLGN where GABAergic cells are relatively homogenous (Jager et al., 2016; Kalish et al., 2018; Leist et al., 2016). For this reason, we next asked whether we could identify novel molecular markers to characterize the heterogeneity of GABAergic neurons in vLGN. We assessed gene expression profiles in the developing mouse vLGN and dLGN in previously generated RNAseq datasets (He et al., 2019; Monavarfeshani et al., 2018). Our rationale was to identify candidate cell type makers by focusing our attention on genes which were: (1) enriched in vLGN but not dLGN, (2) expressed by GABAergic cells in other brain regions, and/or (3) expressed with different developmental patterns which could indicate expression by different subsets of neurons. To characterize if any of these genes labeled distinct populations of neurons in vLGN, we generated >40 riboprobes to perform ISH (Table S1). We also took advantage of available cell type‐specific reporter mice and antibodies for this screen.

From this unbiased riboprobe screen, we identified two genes, *Nxph1* and *Arx*, whose expression in vLGN was restricted to cells in largely non‐overlapping domains. *Nxph1,* which encodes the α‐Nrxn ligand Neurexophilin‐1, was expressed in the most lateral portion of vLGN (Figure 1j‐k). *Arx,* which encodes the homeobox transcription factor Aristaless Related Homeobox protein, was expressed in the most medial portion of vLGN (Figure 1l‐m). Double‐ISH, with *Gad1* riboprobes, revealed that both *Nxph1* and *Arx* mRNAs were generated by GABAergic cells in vLGN (Figure 1k, m and n–o). To test whether *Nxph1* and *Arx* marked GABAergic cell types in vLGNe and vLGNi, respectively, we labeled retinal ganglion cell arbors in vLGN by intraocular injection of fluorescently conjugated Cholera Toxin Subunit B (CTB) into both eyes (Figure 1p). Indeed, we found that *Nxph1* ^+^ neurons reside in vLGNe and *Arx* ^+^ neurons reside in vLGNi (Figure 1q‐r). These results further suggested that transcriptionally distinct subsets of GABAergic neurons exist in vLGN and demonstrated cellular diversity in both laminae of vLGN—vLGNe and vLGNi. Notably, the expression of these two molecules differed in the intergeniculate leaflet (IGL)—a slice of retinorecipient tissue separating vLGN from dLGN which contains a similar proportion of GABAergic neurons to the vLGN, and is often grouped with vLGN because of other shared features (Monavarfeshani et al., 2017). The expression of *Nxph1* in both vLGNe and IGL, and the absence of *Arx* mRNA from IGL, suggests that IGL neurons share similar characteristics with some GABAergic neurons in vLGNe but not vLGNi.

**FIGURE 2:**
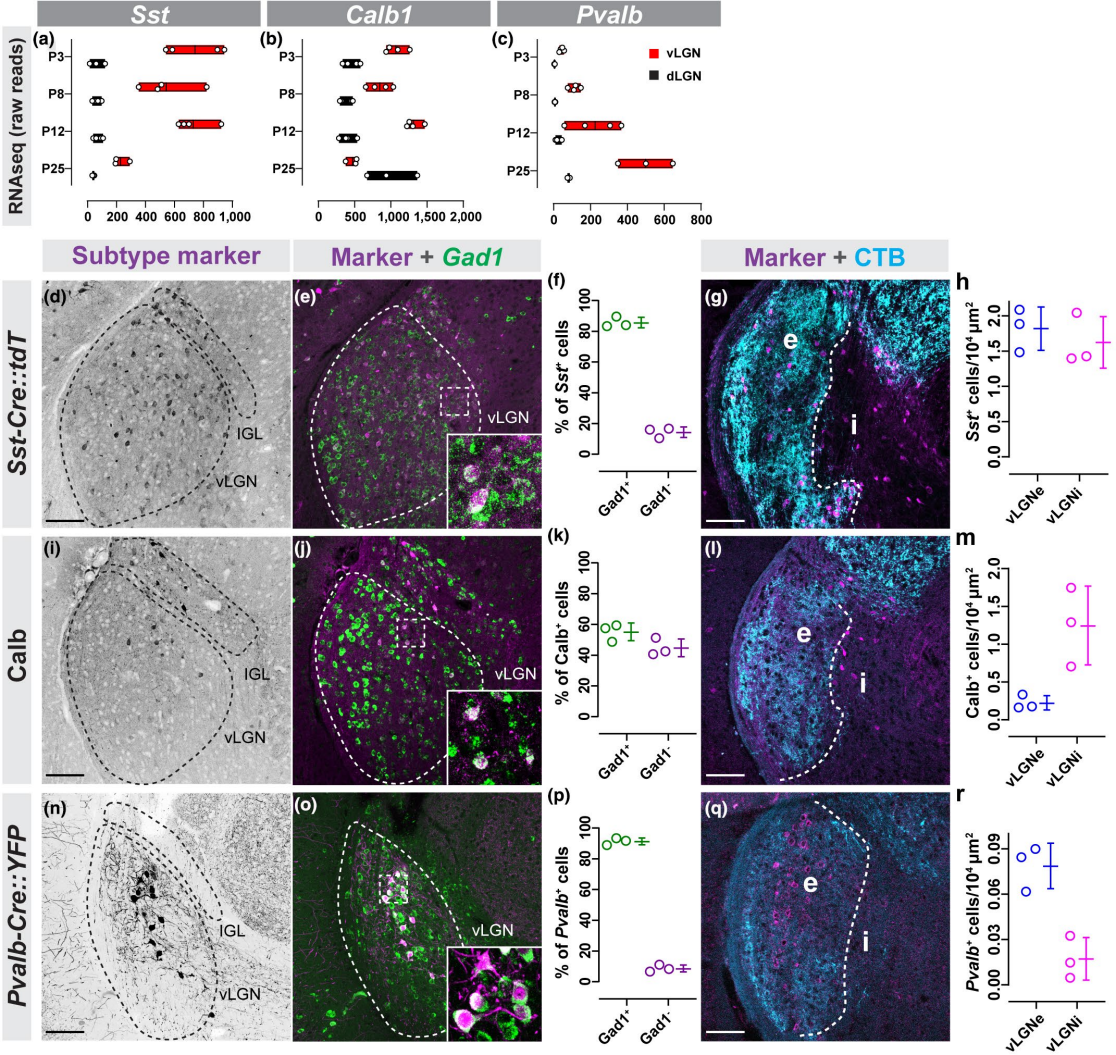
Canonical markers of GABAergic neurons label subsets of neurons in vLGN. (a–c) Raw transcript reads *Sst* (a)*, Calb1* (b)*, and Pvalb* (c) mRNA in vLGN and dLGN obtained by RNAseq. Individual data points plotted as white circles, min/max values are confined to the red bars, and vertical black line with bars depicts mean. (d) Transgenic labeling of *Sst* ^+^ neurons in vLGN of P60 *Sst‐Cre::Rosa‐Stop‐tdT* mice. (e) In situ hybridization (ISH) for *Gad1* mRNA in *Sst‐Cre::Rosa‐Stop‐tdT* vLGN. (f) Quantification of the percentage of *Sst* ^+^ cells that co‐express *Gad1* mRNA. Data points represent biological replicates, bars represent mean ± *SD*. (g) Transgenic labeling of *Sst* ^+^ neurons in vLGNe and vLGNi of *Sst‐Cre:Rosa‐Stop‐tdT* mice following intravitreal cholera toxin subunit b (CTB) injection. (h) Quantification of the density of transgenically labeled *Sst* ^+^ cells in vLGNe and vLGNi. Data plotted as in (f). (i) Immunolabeling of Calb^+^ cells in P60 vLGN. (j) IHC‐ISH for Calb protein and *Gad1* mRNA. (k) Quantification of Calb^+^ and *Gad1* ^+^ signal colocalization as seen in (j). Data plotted as in (f). (l) Calb^+^ neurons in vLGNe and vLGNi visualized by Calb‐immunolabeling and intravitreal CTB injection. (m) Quantification of the density of Calb‐immunoreactive cells in vLGNe or vLGNi. Data plotted as in (f). (n) Transgenic labeling of *Pvalb* ^+^ neurons in P60 vLGN of P60 *Pvalb‐Cre::Thy1‐Stop‐YFP* mice. (o) ISH for *Gad1* mRNA in *Pvalb‐Cre::Thy1‐Stop‐YFP* vLGN. (p) Quantification of the percentage of *Pvalb* ^+^ cells that co‐express *Gad1* mRNA. Data plotted as in (f). (q) Immunolabeling of Pvalb^+^ neurons in vLGNe and vLGNi of wild‐type mouse following intravitreal CTB injection. (r) Quantification of the density of Pvalb^+^ cells density in vLGNe and vLGNi. Data plotted as in (f). *n* = 3 animals in all data presented. All scale bars = 100 µm

We next sought to determine whether GABAergic neurons residing within vLGNe or vLGNi could be further subdivided into distinct neuronal subtypes. Several of the genes identified as differentially enriched in the vLGN compared to dLGN were canonical markers of inhibitory interneurons in other parts of the brain (i.e., Somatostatin, Calbindin, Parvalbumin) (Figure 2a–c) (Lim, Mi, Llorca, & Marín, 2018; Tremblay, Lee, & Rudy, 2016). To label Somatostatin‐expressing cells, we used two approaches: a *Sst‐Cre::Rosa‐Stop‐tdT* reporter mouse in which all *Sst* ^+^ cells were transgenically labeled with tdT and riboprobes against *Sst* mRNA. Both approaches revealed sparse *Sst* ^+^ cells distributed broadly across both the vLGN and the IGL (Figure 2d and data not shown). By coupling *Gad1* ISH in *Sst‐Cre::Rosa‐Stop‐tdT* tissue, we found that > 85% of *Sst* ^+^ cells generated *Gad1* mRNA and were therefore GABAergic neurons. By labeling retinal axons with CTB and quantifying the density of tdT^+^ cells in vLGNe and vLGNi, we determined that *Sst* ^+^ neurons were evenly distributed in vLGNe or vLGNi (Figure 2e‐h). Next, we immunolabeled Calbindin‐expressing neurons and found that Calb^+^ cells were in both IGL and vLGN, and most were also GABAergic (Figure 2i‐k). When we labeled vLGNe and vLGNi with binocular injections of CTB, we found that Calb^+^ cells were preferentially distributed in vLGNi, although they represent only a fraction of the cells in this region (Figure 2l‐m). Finally, we labeled Parvalbumin‐expressing neurons by generating a *Pvalb‐Cre::Thy1‐Stop‐YFP* mouse in which all *Pvalb* ^+^ cells were transgenically labeled with YFP (Figure 2n). We observed a modest number of *Pvalb* ^+^ cells in vLGN, but unlike the broad distribution of *Sst* ^+^ and Calb^+^ neurons, *Pvalb* ^+^ cells were concentrated in what appeared to be a layer of vLGN and were absent from IGL (Figure 2n). By coupling *Gad1* ISH in *Pvalb‐Cre::Thy1‐Stop‐YFP*, we found that > 90% of *Pvalb* ^+^ cells generated *Gad1* mRNA and were therefore GABAergic (Figure 2o‐p). When we labeled retinal projections in vLGNe with CTB and *Pvalb* ^+^ GABAergic neurons by immunolabeling, we determined that *Pvalb* ^+^ cells were exclusively present in vLGNe (Figure 2q‐r). It was noteworthy that *Sst* ^+^, *Calb1* ^+^, and *Pvalb* ^+^ cells were largely absent from the neighboring dLGN, suggesting that these GABAergic cell types were unique from previously studied dLGN interneurons.

**FIGURE 3:**
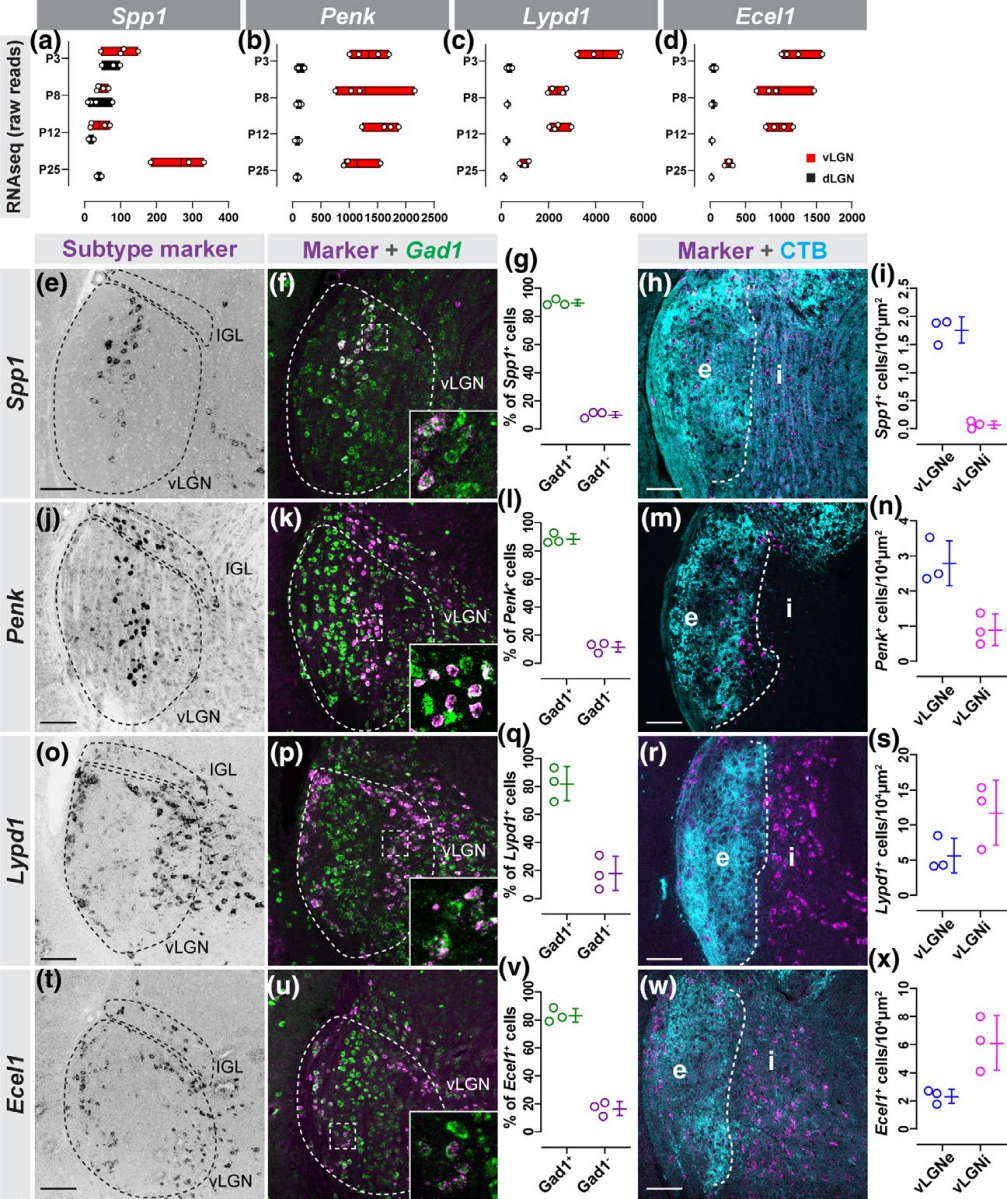
Novel genetic markers label subtypes of GABAergic neurons. (a–d) Raw transcript reads *Spp1* (a)*, Penk* (b)*, Lypd1* (c), and *Ecel1* (d) mRNA in vLGN and dLGN obtained by RNAseq. Individual data points plotted as white circles, min/max values are confined to the red bars, and vertical black line with bars depicts mean. (e) In situ hybridization (ISH) for *Spp1* mRNA in P60 vLGN. (f) Double ISH for *Spp1* and *Gad1* mRNA in vLGN. (g) Quantification of the percentage of *Spp1* ^+^ cells that co‐express *Gad1* mRNA. Data points represent biological replicates, bars represent mean ± *SD*. (h) ISH‐labeling *Spp1* ^+^ neurons in vLGNe and vLGNi following intravitreal cholera toxin subunit b (CTB) injection. (i) Quantification of the density of *Spp1* ^+^ neurons in vLGNe and vLGNi. Data plotted as in (g). (j) ISH for *Penk* mRNA in P60 vLGN. (k) Double ISH for *Penk* and *Gad1* mRNA in vLGN. (l) Quantification of the percentage of *Penk* ^+^ cells that co‐express *Gad1* mRNA. Data plotted as in (g). (m) ISH‐labeling *Penk* ^+^ neurons in vLGNe and vLGNi following intravitreal CTB injection. (n) Quantification of the density of *Penk* ^+^ neurons in vLGNe and vLGNi. Data plotted as in (g). (o) ISH for *Lypd1* mRNA in P10 vLGN. (p) Double ISH for *Lypd1* and *Gad1* mRNA in vLGN. (q) Quantification of the percentage of *Lypd1* ^+^ cells that co‐express *Gad1* mRNA. Data plotted as in (g). (r) ISH‐labeling *Lypd1* ^+^ neurons in vLGNe and vLGNi following intravitreal CTB injection. (s) Quantification of the density of *Lypd1* ^+^ neurons in vLGNe and vLGNi. Data plotted as in (g). (t) ISH for *Ecel1* mRNA in P60 vLGN. (u) Double ISH for *Ecel1* and *Gad1* mRNA in vLGN. (v) Quantification of the percentage of *Ecel1* ^+^ cells that co‐express *Gad1* mRNA. Data plotted as in (g). (w) ISH‐labeling *Ecel1* ^+^ neurons in vLGNe and vLGNi following intravitreal CTB injection. (x) Quantification of the density of *Ecel1* ^+^ neurons in vLGNe and vLGNi. Data plotted as in (g). *n* = 3 animals in all data presented. All scale bars = 100 µm

Since neither the *Pvalb* ^+^ neurons (which preferred the vLGNe) nor Calb^+^ neurons (which preferred the vLGNi) labeled all of the cells in their respective subdomains, we hypothesized that an even richer heterogeneity of GABAergic cells existed. This led us to ask whether there were other types of GABAergic neurons which exhibited similar vLGN subdomain preferences. Using our riboprobe screen, we identified four additional genes that were generated by regionally restricted subsets of cells in vLGN: *Spp1*, *Penk*, *Lypd1*, and *Ecel1*. The transcripts for all of these genes were enriched in vLGN compared to dLGN (Figure 3a‐d) and were generated by *Gad1* ^+^ GABAergic neurons (Figure 3e‐x).

Riboprobes generated against *Spp1*, which encodes the extracellular glycoprotein Osteopontin, revealed *Spp1* ^+^ cells were sparsely present in the vLGN (and absent from both IGL and dLGN). Interestingly, *Spp1* ^+^ cells were distributed in a stratified fashion within vLGNe, just as we observed for *Pvalb* ^+^ cells (Figure 3e,h‐i). ISH for *Penk,* which encodes Proenkephalin, revealed that *Penk* mRNA was present in a subset of vLGN cells and in many IGL neurons (Figure 3j‐k). Like what we observed for *Spp1* ^+^ and *Pvalb* ^+^ neurons, *Penk* ^+^ neurons also appeared distributed in a stratified fashion. Labeling retinal afferents with CTB revealed that both *Spp1* ^+^ and *Penk* ^+^ neurons were located in the retinorecipient vLGNe, although it was unclear if they were present in the same region (Figure 3m‐n). Finally, ISH for *Lypd1,* which encodes LY6/PLAUR Domain Containing 1, and *Ecel1*, which encodes Endothelin Converting Enzyme Like 1, revealed that these genes exhibited similar cellular expression patterns in vLGN and IGL (Figure 3o,t). *Lypd1* ^+^ and *Ecel1* ^+^ cells were sparsely distributed in the IGL and densely populated two distinct and separate regions of the vLGN, occupying both the lateral‐most region of vLGNe and the entire vLGNi (Figure 3r‐s,w‐x). Based on similarities of expression patterns in both vLGN and IGL, it seemed likely that *Ecel1* and *Lypd1* mRNAs were generated in the same subsets of GABAergic neurons. Notably, our analyses of vLGN neuronal subtypes once again indicate that some types of vLGNe cells (such as *Penk* ^+^ neurons) may be present in IGL, but some types (such as *Pvalb* ^+^ and *Spp1* ^+^ *neurons*) are absent from IGL.

Taken together, these experiments reveal novel markers of transcriptomically and regionally distinct subsets of GABAergic neurons in vLGN. Importantly, not only did these cells have regional preferences, but *Pvalb* ^+^, *Penk* ^+^, *Spp1* ^+^, *Ecel1* ^+^, and *Lypd1* ^+^ neurons each appeared to be organized into segregated strata that span the dorso‐ventral axis of the vLGN.

### 3.2 Transcriptomically distinct GABAergic neurons organize into adjacent sublaminae of vLGNe

**FIGURE 4:**
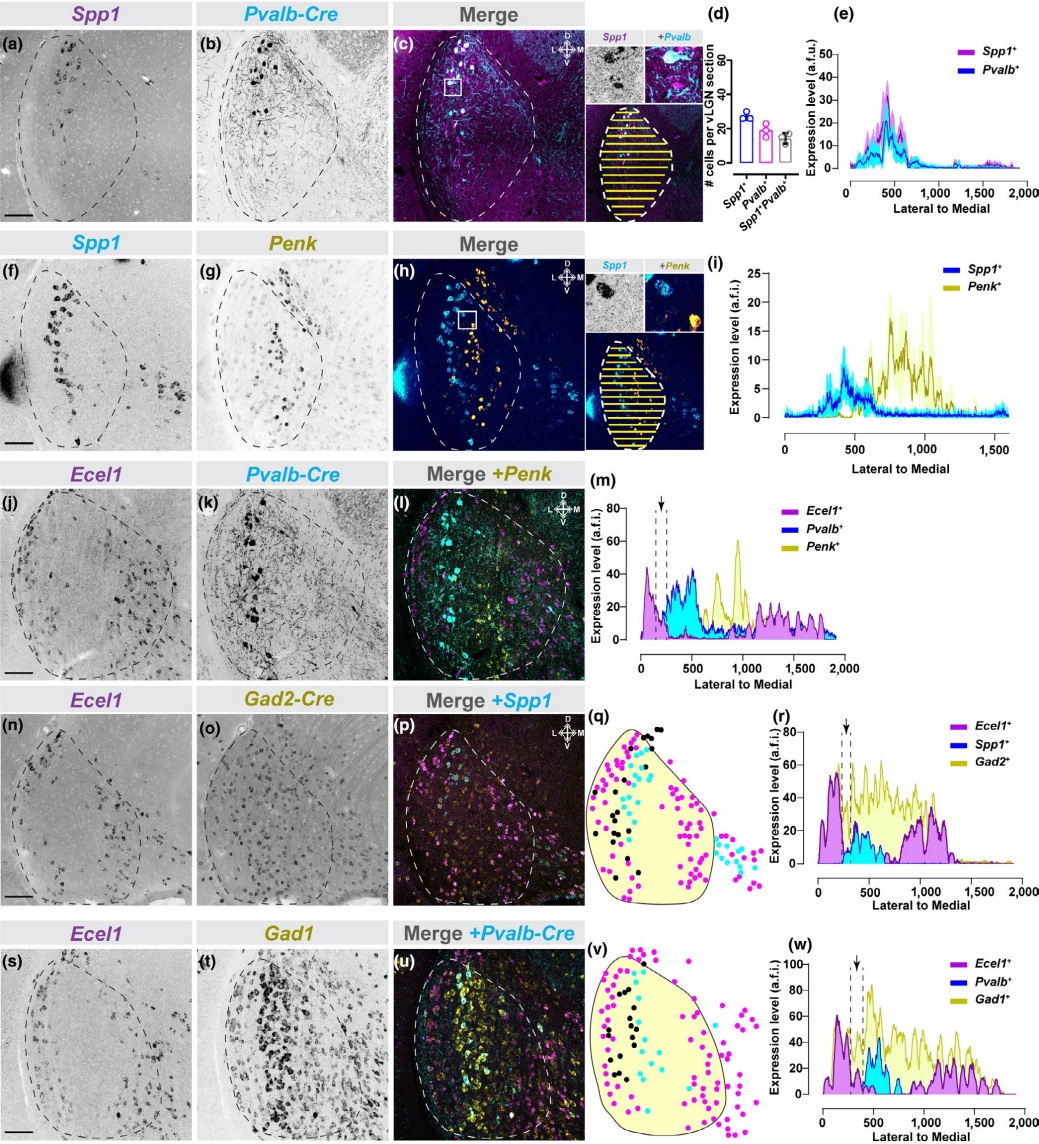
Transcriptomically distinct GABAergic neurons organize into discrete sublaminae of vLGNe. (a–c) In situ hybridization (ISH) ‐labeling for Spp1^+^ neurons in vLGN of P60 *Pvalb‐Cre::Thy1‐Stop‐YFP* transgenic reporter mice. Inset in (c) shows single channel and merged hi‐magnification images and illustrates automated line scan approach used to quantify spatial expression. (d) Quantification of vLGN neurons which generate either or both *Spp1* and *Pvalb* mRNA. Data points represent biological replicates, bars represent mean ± *SD*. (e) Line scan analysis of spatial distribution of *Spp1* ^+^ and *Pvalb^+^* cells. Arbitrary fluorescence units (a.f.i.) are plotted against distance from the lateral‐most part of the vLGN to the most medial. Solid line represents mean and shaded area represents *SEM* (*n* = 3 animals). (f–h) Double ISH for *Spp1* and *Penk* mRNA in P60 vLGN. Inset in (h) shows single channel and merged hi‐magnification images and illustrates automated line scan approach used to quantify spatial expression. (i) Line scan analysis of spatial distribution of *Spp1^+^* and *Penk^+^* cells in vLGN plotted as in (e), *n* = 3 animals. (j–l) Double ISH for *Ecel1* and *Penk* mRNA in vLGN of P60 *Pvalb‐Cre::Thy1‐Stop‐YFP* transgenic reporter mice. (m) Line scan analysis of spatial distribution of *Ecel1^+^*, *Penk^+^*, and *Pvalb^+^* cells in vLGN, plotted as in (e), revealing four discrete spatial domains of vLGN. The black arrow is pointing at a potential fifth layer between *Ecel1^+^* and *Pvalb^+^* layers. (n–p) Double ISH for *Ecel1* and *Spp1* mRNA in vLGN of P60 *Gad2‐Cre::Sun1‐Stop‐GFP* transgenic reporter mice. (q) Schematic of subtype expression observed in (p), where each dot corresponds to a cell (magenta = *Ecel1^+^*, cyan = *Spp1^+^*, black = *Ecel1^‐^Spp1^‐^Gad2^+^*). Only *Gad2^+^* neurons between the two labeled layers are identified by black dots. (r) Line scan analysis of spatial distribution of *Ecel1^+^*, *Spp1^+^*, and *Gad2^+^* cells in vLGN, plotted as described in (e). Black arrow points at same region as arrow in (m). (s–u) Double ISH for *Ecel1* and *Gad1* mRNA in vLGN of P60 *Pvalb‐Cre::Thy1‐Stop‐YFP* transgenic reporter mice. (v) Schematic of subtype expression observed in (u), where each dot corresponds to a cell (magenta = *Ecel1^+^*, cyan = *Pvalb^+^*, black = *Ecel1^‐^Pvalb^‐^Gad1^+^ neurons*), as in (q). (w) Line scan analysis of spatial distribution of *Ecel1^+^*, *Pvalb^+^*, and *Gad1^+^* cells in vLGN, plotted as in (e). Black arrow points at same region as arrow in (m and r). *n* = 3 animals in d,e,i and *n* = 1 animal in m,r,w. All scale bars = 100 µm

We next asked whether these were in fact mutually exclusive groups of cells and whether they corresponded to distinct vLGNe sublaminae. We started by assessing whether *Spp1* and *Pvalb* mRNAs were generated by the same neurons or occupied the same dorso‐ventral zone. Performing ISH on *Pvalb‐Cre::Thy1‐Stop‐YFP* tissue revealed that *Spp1* mRNA was generated by some, but not all, *Pvalb* ^+^ cells (~50% *Spp1* ^+^ neurons were *Pvalb^‐^*) and vice versa (~25% *Pvalb* ^+^ neurons were *Spp1^‐^*)(Figure 4a–d). *Spp1* ^+^ *Pvalb* ^+^, *Spp* ^+^ *Pvalb^‐^*, and *Pvalb^‐^Spp1* ^+^ cells all appeared to reside within the same sublamina of vLGNe. To quantitatively assess cellular distribution in vLGN, we developed an automated script in ImageJ to measure fluorescent intensity along the medial‐lateral axis of vLGN (Figure 4c inset). Fluorescent signals at each medial‐lateral coordinate were averaged along the entire dorsoventral extent of vLGN (and between biological replicates) and the quantified data identified the same spatial region of vLGN as populated both *Spp1* ^+^ and *Pvalb* ^+^ cells (Figure 4e).

Next, we asked whether *Penk* was generated by either *Spp1* ^+^ or *Pvalb* ^+^ cells. For this, we used double ISH or genetic reporter lines. In both cases, we were unable to identify a single occurrence in which *Penk* ^+^ neurons co‐expressed either *Pvalb* or *Spp1* (Figure 4f–h and data not shown). Moreover, these experiments clearly demonstrated that *Penk* ^+^ cells were not only transcriptomically distinct from *Spp1* ^+^ and *Pvalb* ^+^ cells, but also were present in an adjacent sublamina. Line scan analysis of fluorescence from *Penk* and *Spp1* double ISH experiments confirmed that these populations of GABAergic neurons were present in distinct sublaminae (Figure 4i). Next, we took a triple labeling approach (double ISH for *Ecel1* ^+^ and *Penk* ^+^ neurons in the transgenic *Pvalb‐Cre::Thy1‐Stop‐YFP*) to test whether the sublaminae populated by *Pvalb* ^+^ and *Penk* ^+^ cells were distinct from those populated by *Ecel1* ^+^ cells (Figure 4j–m). Again, we observed no *Ecel1* ^+^ *Pvalb* ^+^ or *Ecel1* ^+^ *Penk* ^+^ neurons using this method, and quantitative analysis of *Ecel1/Penk/Pvalb* expression patterns along the medio‐lateral extent of vLGN revealed at least three distinct sublaminae in vLGNe (Figure 4i).

Labeling of *Ecel1* ^+^, *Pvalb* ^+^, and *Penk* ^+^ cells at once did not label all GABAergic cells in vLGN. In fact, it appeared as if the space between the lateral‐most layer of *Ecel1* ^+^ cells and the *Spp1* ^+^ layer may represent another layer of GABAergic cells which we failed to identify with our riboprobe screen (arrow in Figure 4m). To test this idea, we performed two triple labeling experiments: one in which we labeled *Ecel1* and *Spp1* mRNA in *Gad2‐Cre::Sun1‐Stop‐GFP* tissue, and one in which we labeled *Ecel1* and *Gad1* mRNAs in *Pvalb‐Cre::Thy1‐Stop‐YFP* tissue (Figure 4n–w). In both cases, we identified GABAergic cells between the *Ecel1* ^+^ layer and the *Spp1* ^+^ *Pvalb* ^+^ layer. Line scan analysis confirmed a small population of *Gad2* ^+^ *Ecel1* ^‐^ *Spp1* ^‐^ neurons between the sublaminae containing *Ecel1* ^+^ or *Spp1* ^+^ neurons (Figure 4q–r, v–w), suggesting the existence of at least a fourth sublamina in vLGNe with yet‐to‐be‐defined GABAergic neurons.

**FIGURE 5:**
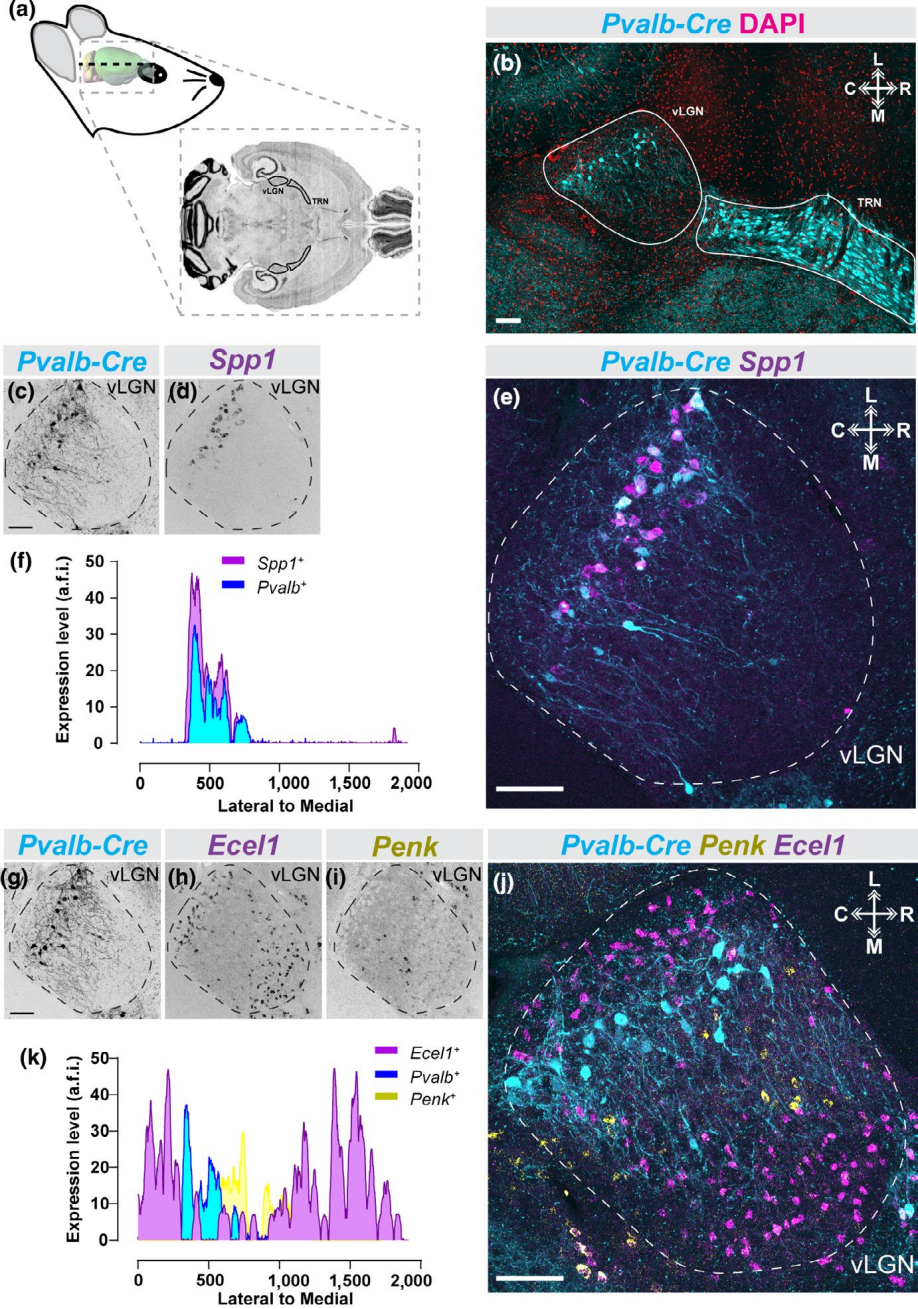
Subtype‐specific laminar organization of vLGN neurons along the rostro‐caudal axis. (a) Schematic of horizontal mouse brain sectioning used to find vLGN. (b) Identification of the mouse vLGN and thalamic reticular nucleus (TRN) in the horizontal plane using the *Pvalb‐Cre::Thy1‐Stop‐YFP* transgenic reporter and 4′,6‐diamidino‐2‐phenylindole (DAPI) counter‐staining. (c–e) in situ hybridization (ISH) ‐labeling for *Spp1^+^* neurons in horizontal vLGN of P60 *Pvalb‐Cre::Thy1‐Stop‐YFP* transgenic reporter mice. (f) Line scan analysis of spatial distribution of *Spp1^+^* and *Pvalb^+^* cells. Arbitrary fluorescence units (a.f.i.) are plotted against distance from the lateral‐most part of the vLGN to the most medial. (g–j) Double ISH for *Ecel1* and *Penk* mRNA in horizontal vLGN of P60 *Pvalb‐Cre::Thy1‐Stop‐YFP* transgenic reporter mice. (k) Line scan analysis of spatial distribution of *Ecel1^+^*, *Penk^+^*, and *Pvalb^+^* cells in vLGN, plotted as in (f), revealing four discrete spatial domains of vLGN. *n* = 1 animal for all data presented. All scale bars = 100 µm

We recognized that our spatial registration of GABAergic subtypes into distinct sublaminae in vLGNe might have been an artifact of the coronal plane of section. To determine whether these sublaminae were in fact true structural components of vLGN, we performed axial (horizontal) sections of *Pvalb‐Cre::Thy1‐Stop‐YFP* (Figure 5a and b). By performing double ISH on this tissue, we found that (1) *Spp1* ^+^ and *Pvalb* ^+^ cells reside in the same layer (and a population of cells express both transcripts), and (2) *Ecel1* ^+^, *Pvalb* ^+^, and *Penk* ^+^ cells populate distinct sublaminae of vLGNe (Figure 5c‐k). Taken together, these data demonstrate, for the first time, that the vLGNe contains heterogeneous populations of transcriptomically distinct GABAergic cell types that map onto at least four adjacent sublaminae (that are not appreciable with conventional histochemical staining approaches).

### 3.3 GABAergic neurons in the four sublaminae of vLGNe are retinorecipient

**FIGURE 6:**
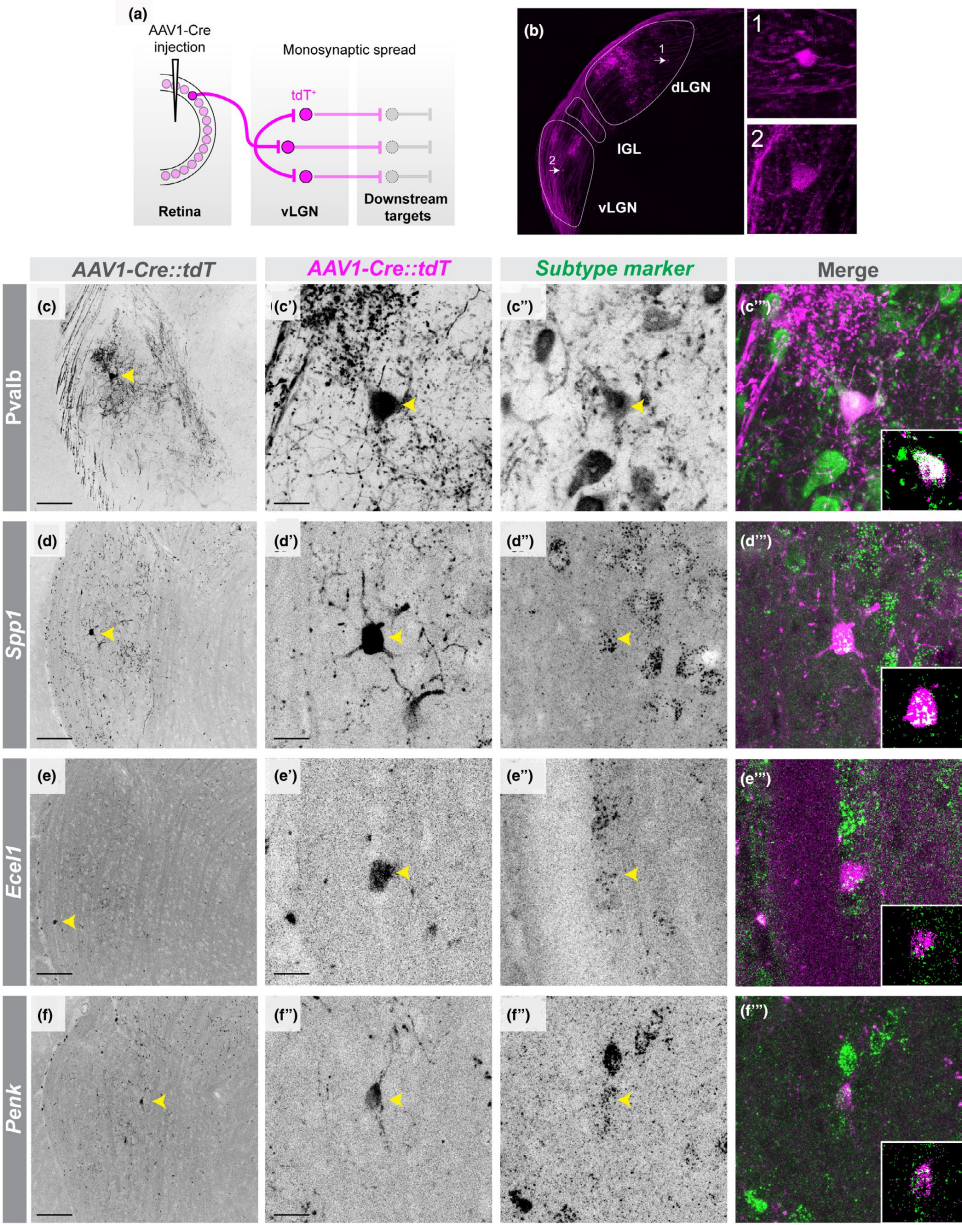
Several subtypes of GABAergic neurons in vLGNe receive direct retinal input. (a) Schematic of trans‐synaptic viral tracing strategy to label retinorecipient vLGN neurons, where AAV1‐Cre induces recombination (and therefore expression of tdT) in transfected cells. (b) Image of an LGN with retinorecipient cells labeled using strategy in (a). (c‐c’) low‐magnification and high‐magnification micrographs, respectively, of labeled retinorecipient neuron in vLGN. (c’’‐c’’’) Immunolabeling of Pvalb^+^ neurons in vLGN (c’’) and colocalization with tdT^+^ signal (c’’’). (d‐d’) low‐magnification and high‐magnification micrographs, respectively, of labeled retinorecipient neuron in vLGN. (d’’‐d’’’) in situ hybridization (ISH) labeling of *Spp1^+^* neurons in vLGN (d’’) and colocalization with tdT^+^ signal (d’’’). (e‐e’) low‐magnification and high‐magnification micrographs, respectively, of labeled retinorecipient neuron in vLGN. (e’’‐e’’’) ISH labeling of *Ecel1^+^* neurons in vLGN (e’’) and colocalization with tdT^+^ signal (e’’’). (f‐f’) low‐magnification and high‐magnification micrographs, respectively, of labeled retinorecipient neuron in vLGN. (f’’‐f’’’) ISH labeling of *Penk^+^* neurons in vLGN (f’’) and colocalization with tdT^+^ signal (f’’’). Insets are single plane confocal images. *n* = 1 animal for all data presented. Scale bars (c,d,e,f) = 100 µm, (c’,d’,e’,f’) = 25 µm

The laminar segregation of transcriptomically distinct cell types in vLGN raises the possibility that this organization functions to parse different streams of visual information. This led us to hypothesize that GABAergic cells in distinct sublaminae in vLGNe were directly innervated by retinal axons. In fact, while retinorecipient cells in dLGN are well defined, the retinorecipient neurons in vLGN are largely unknown. To anterogradely label retinorecipient cells in vLGN, we intravitreally injected a trans‐synaptic adeno‐associated virus expressing Cre recombinase (AAV2/1‐hSyn‐Cre‐WPRE‐hGH, referred to here as AAV1‐Cre) into *Rosa‐Stop‐tdT* mice (Zingg et al., 2017) (Figure 6a and b). This trans‐synaptic viral transfection strategy has previously been shown to accurately map monosynaptic connections and, in our hands, resulted in sparse tdT^+^ labeling of cells in retinorecipient brain regions (Figure 6b) (Zingg et al., 2017). Using this approach, we trans‐synaptically labeled 53 retinorecipient neurons in vLGN (*n* = 12 animals). Once we identified tdT^+^ cells in vLGN, we assessed their spatial localization relative to the sublaminae described earlier and performed ISH to determine whether the distinct subtypes of GABAergic neurons identified here were retinorecipient. We identified *Pvalb* ^+^, *Spp1* ^+^, *Ecel1* ^+^, and *Penk* ^+^ cells in vLGNe that contained tdT, indicating that these populations of GABAergic neurons are capable of receiving monosynaptic input from the retina (Figure 6c–f).

**FIGURE 7:**
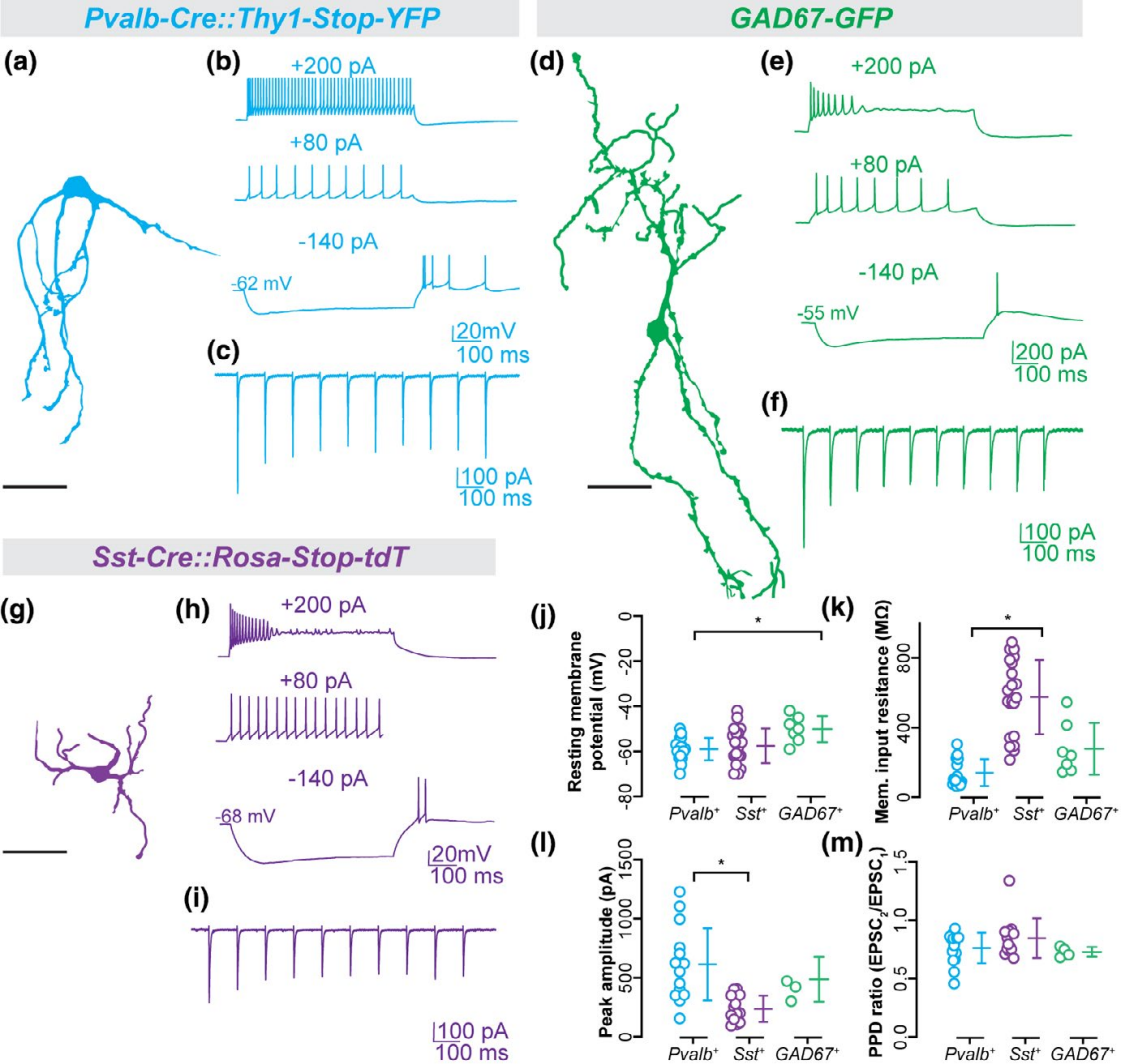
Morphology, synaptic responses, and membrane properties of GABAergic subtypes in vLGNe. (a–i) Representative confocal reconstructions of *Pvalb^+^* (a), *GAD67^+^* (d), and *Sst^+^* (g) neurons along with examples of their voltage responses to hyperpolarizing and depolarizing current pulses (top), and synaptic responses to optic tract (OT) stimulation (bottom). (j) Plot depicting resting membrane potential of *Pvalb^+^* (*n* = 18 cells), *Sst^+^* (*n* = 22 cells), and *GAD67^+^* neurons (*n* = 7 cells). (k) Plot depicting membrane input resistance of *Pvalb^+^* (*n* = 18 cells), *Sst^+^* (*n* = 22 cells), and *GAD67^+^* neurons (*n* = 7 cells). (l) Plot depicting peak excitatory post‐synaptic currents (EPSC) amplitude of *Pvalb^+^* (*n* = 15 cells), *Sst^+^* (*n* = 13 cells), and *GAD67^+^* neurons (*n* = 4 cells). (m) Plot depicting paired pulse depression ratio (PPD) during repeated stimulation of *Pvalb^+^* (*n* = 15 cells), *Sst^+^* (*n* = 13 cells), and *GAD67^+^* neurons (*n* = 4 cells). Each data point represents an individual value and bars reflect mean ± *SEM*. Asterisks and bars indicate statistically significant (*p* < .05) determined by a Kruskal–Wallis test with Dunn's correction for multiple comparisons. All scale bars = 50 µm

We next sought to functionally confirm that some of these transcriptomically distinct cell types receive direct retinal input and to characterize their electrophysiological response properties. We utilized available transgenic reporter lines to record from and characterize several GABAergic subtypes in vLGN. Figure 7a–c provide examples of biocytin filled vLGN neurons recorded from *Pvalb‐Cre::Thy1‐Stop‐YFP* (*n* = 12), *Sst‐Cre::Rosa‐Stop‐tdT* (*n* = 15) and *GAD67‐GFP* (*n* = 5) mice along with representative voltage responses to current injection and synaptic responses evoked by optic tract (OT) stimulation. Overall, their dendritic architecture varied widely from each other. *Pvalb* ^+^ neurons had a hemispheric architecture with several elongated but sparsely branched primary dendrites, whereas *Sst* ^+^ neurons had much smaller bipolar dendritic fields that contained short multi‐branched processes. Biocytin filled vLGN neurons from *GAD67‐GFP* mice had similar morphologies to intrinsic interneurons in dLGN, displaying expansive and complex arbors that arise from opposite poles of fusiform‐shaped soma (Charalambakis et al., 2019; Guillery, 1966; Seabrook et al., 2013). The voltage responses to current injection also revealed differences in their intrinsic membrane and firing properties. For example, the resting membrane potential (Figure 7j) of *Pvalb* ^+^ (−59 ± 1.2 mV; *n* = 18) neurons was significantly (Kruskal–Wallis H = 8.080, *p* = .0149, Dunn's multiple comparisons) hyperpolarized compared to *GAD67‐GFP* ^+^ neurons (−50 ± 2.2 mV; *n* = 7). The input resistance (Figure 7k) of *Pvalb* ^+^ (140 ± 18 MΩ; *n* = 18) was also significantly (Kruskal–Wallis H = 29.66, *p* < .0001, Dunn's multiple comparisons) lower compared to *Sst* ^+^ (575 ± 46 MΩ; *n* = 22). Responses to hyperpolarizing current pulses failed to evoke a large triangular shaped, rebound low threshold Ca^2+^ spike (Figure 7b,e and h). However, both *Sst* ^+^ and *GAD67‐GFP* ^+^ neurons showed a strong depolarizing sag which is likely mediated by hyperpolarization activated cation conductance (Ih), whereas *Pvalb* ^+^ neurons showed little or no such inward rectification. For all three cell types, small depolarizing current steps (~80 pA) evoked a train of spikes that displayed spike frequency adaptation. However, when larger steps were employed (> 100 pA), *Pvalb* ^+^ neurons displayed high frequency firing (84 ± 18 Hz). However, *Sst* ^+^ and *GAD67‐GFP* ^+^ neurons had difficulty maintaining firing throughout the duration of the current step, showing a progressive inactivation of Na^2+^ spikes. Analyses of morphology, resting membrane potential, and input resistance indicate that *Pvalb* ^+^, *Sst* ^+^, and *GAD67‐GFP* ^+^ neurons are not only transcriptionally distinct subtypes of cells (i.e., t‐types (Scala, Kobak, & Bernabucci, 2020)), but also are distinct cell types based on morphology and electrophysiological properties (i.e., morpho‐electric types [me‐types] (Gouwens et al., 2019)) (Figure 7j,k).

Finally, we examined the synaptic responses that were evoked by OT stimulation (Figure 7c, f and i). As expected, OT stimulation evoked excitatory post‐synaptic current activity in neurons (38/46, 82.6%) located within vLGNe, whereas those recorded (*n* = 14) in vLGNi failed to respond. The high prevalence of synaptic responses in the vLGNe was observed among *Pvalb* ^+^ (18/19), *Sst* ^+^ (13/15) and *GAD67‐GFP* ^+^ (4/7) neurons. The peak amplitude of excitatory currents (Figure 7l) for *Pvalb* ^+^ (613 ± 79 pA; *n* = 15) neurons was significantly (Kruskal–Wallis H = 14.55, *p* = .0006, Dunn's multiple comparisons) higher compared to *Sst* ^+^ neurons (237 ± 31; *n* = 13). Synaptic responses evoked by a 10Hz stimulus train showed a moderate depression (75%–85%) after the initial response. Measurements of paired pulse depression (Figure 7m) revealed no significant (Kruskal–Wallis H = 3.123, *p* > .1) differences between *Pvalb* ^+^ (0.76 ± 0.03; *n* = 15), *Sst* ^+^ (0.84 ± 0.04; *n* = 13) and *GAD67‐GFP* ^+^ (0.7 ± 0.02; *n* = 4) neurons. These results demonstrate two important points. First, all three of these distinct GABAergic cell types receive direct input from RGCs (that is likely to be a driver‐like input, based on the presence of paired pulse depression; (Petrof & Sherman, 2013)). Second, the strength of retinal inputs onto some of these cell types differed, suggesting that there may be unique anatomical properties or distributions of retinal synapses onto GABAergic neurons in vLGN.

## 4 DISCUSSION

In this study, we identified novel vLGN cell‐type markers which label GABAergic cells throughout the nucleus and distinguish it from its dorsal counterpart—the dLGN. In some cases, these novel GABAergic cell types also distinguished vLGN from IGL, although some transcriptionally defined cell types also appear to be shared between these two regions. By performing a series of multiplex labeling experiments using these newly identified markers, we revealed a remarkably organized laminar architecture of vLGNe, composed of at least four adjacent, transcriptionally distinct sublaminae. Interestingly, such organization was absent from IGL, even for cell types present in both regions. Using anterograde trans‐synaptic viral tracing and patch‐clamp electrophysiology, we determined that many of these regionally and transcriptomically distinct subtypes of GABAergic neurons receive direct retinal input. Thus, these data reveal a novel cellular organization of the vLGN and suggest such organization may have important implications for how different streams of retina‐derived visual information are processed in this part of visual thalamus.

### 4.1 Different types of hidden laminae exist in vLGN and dLGN

In contrast to the clear lamination of the primate and carnivore lateral geniculate nucleus, the rodent dLGN and vLGN have no obvious cytoarchitectonic lamination—save for the division of the vLGN into retinorecipient vLGNe and non‐retinorecipient vLGNi (Gabbott & Bacon, 1994; Harrington, 1997; Hickey & Spear, 1976; Niimi et al., 1963; Sabbagh et al., 2018). The neuronal cytoarchitecture of the rodent dLGN is composed of three well‐defined classes of glutamatergic thalamocortical relay cells and just one or two classes of GABAergic inhibitory interneurons (roughly 10% of its neuronal population) (Arcelli et al., 1997; Evangelio et al., 2018; Jaubert‐Miazza et al., 2005). While interneurons are present throughout the nucleus, relay cells of each class appear to exhibit regional preferences in their distribution within the dLGN (Krahe et al., 2011). These regional preferences in the rodent dLGN, however, do not alone capture the level of organization seen in primate and carnivore LGN. Instead, it appears that retinal afferents impart order in the rodent dLGN by segregating their arbors into “hidden laminae” both in terms of eye‐specific domains and subtype‐specific lamina (Hong & Chen, 2011; Martin, 1986; Reese, 1988). Recent advances in transgenic labeling of individual RGC subtypes has revealed the precise architecture of these hidden laminae of subtype‐specific retinal arbors in dLGN (Cruz‐Martín et al., 2014; Huberman et al., 2008, 2009; Kay et al., 2011; Kerschensteiner & Guido, 2017; Kim et al., 2008, 2010; Martersteck et al., 2017; Monavarfeshani et al., 2017; Rivlin‐Etzion et al., 2011). In this study, however, we identified a different kind of ‘hidden laminae’ within vLGN. Notably, the few identified subtypes of vLGN‐projecting RGCs do not appear to segregate their terminal arbors into laminae in vLGN (with the notable exception that they are restricted to vLGNe) (Hattar et al., 2006; Monavarfeshani et al., 2017; Osterhout et al., 2011). The diffuse terminal arborization of these non‐image forming subtypes of RGCs raises the question of whether visual information is, in fact, parsed into parallel channels in vLGN (Hattar et al., 2006; Osterhout et al., 2011). The stratification of transcriptomically distinct neurons presented in this study into adjacent sublaminae in vLGNe may contribute to the parallel processing of sensory information in this brain region. Just as the organization of retinal inputs imposes order on the otherwise less organized cytoarchitecture of dLGN, the diversity and organization of intrinsic cells in vLGN may impose order on the unorganized input it receives from the retina.

### 4.2 Do laminated retinorecipient circuits organize visual pathways through the vLGN?

Is the quantifiable segregation of distinct GABAergic subtypes into sublaminae a potential means of organizing visual information arriving from the retina? To address this, we sought to determine whether these subtypes were directly innervated by RGCs. Using anterograde trans‐synaptic tracing, we identified *Ecel1* ^+^, *Pvalb* ^+^, *Spp1* ^+^, and *Penk* ^+^ cells as retinorecipient, together representing at least three sublaminae of vLGNe. While we failed to observe any retinorecipient cells in vLGNi using this method, it certainly remains possible that the dendrites of cells in vLGNi extend into vLGNe and receive monosynaptic input from the retina. Nevertheless, our viral tracing results suggest that the cell‐type‐specific organization of the vLGN is relevant to organizing visual input. Such organization of retinorecipient cells hints at a potentially novel role for vLGN in visual processing, by which incoming visual input is sampled by specific GABAergic cell types and parsed into parallel channels of sensory information to be transmitted to downstream targets.

The specificity of these organized cell types in vLGN raises questions of whether they are projection neurons or local interneurons. Our electrophysiological and morphological analyses of some of these cell types suggests that cells labeled in *GAD67‐GFP* mice are likely vLGN interneurons. Based on their preponderance in the vLGN, it is likely that at least some of the other subtypes of GABAergic neurons here are projection neurons. Unlike the dLGN, which has afferent projections to just visual cortex and the thalamic reticular nucleus, neurons in vLGN project to a diverse set of over a dozen downstream subcortical regions including the SC, the nucleus of the optic tract and other pretectal nuclei (Cadusseau & Roger, 1991; Swanson, Cowan, & Jones, 1974; Trejo & Cicerone, 1984), the suprachiasmatic nucleus (at least in hamsters)(Harrington, 1997), the habenula (Huang et al., 2019; Oh et al., 2014), and zona incerta (Ribak & Peters, 1975), all contributing to visuomotor, ocular, vestibular, circadian, and other innate behaviors (Monavarfeshani et al., 2017). It is known that some GABAergic cells in vLGN receive retinal input and project to the lateral habenula, although it remains unclear which GABAergic subtypes this includes (Huang et al., 2019).

*Might the different transcriptionally distinct GABAergic cell types in vLGN each project to different downstream nuclei and contribute to unique functions and behaviours?* First, we note here that it is conceivable that vLGN not only organizes and transmits visual information in separate channels, but also samples specific features of retinal input in sublamina‐specific manner—consistent with labeled‐line theory. Unfortunately, we do not yet have subtype specific resolution of vLGN projection neurons but hope that the data presented here will help to create a molecular toolkit for such circuit tracing in future studies. Such experiments will be crucial in a) determining whether parsing visual information into these hidden laminae is important for parallel processing and b) whether there is a functional and/or projection‐specific logic to the lamination of vLGN cell types.

### 4.3 Transcriptomic heterogeneity underlying cellular diversity in vLGN

There is a current push to use unbiased approaches to identify all of the cell types in the brain—essentially to create a “parts” list. These studies have typically employed single cell RNA sequencing (scRNAseq) to understand the development, structure, and evolution of the brain (Krienen, Goldman, & Zhang, 2019; Peng et al., 2019; Saunders et al., 2018). In a neuroscience community that consists of “lumpers” and “splitters,” it is clear we are currently in an era of “splitting”—as our technology to detect transcriptomic heterogeneity of cell types evolves, so too does the granularity of their discretization. Here, we have begun to “split” the rodent vLGN into many distinct GABAergic cell types.

Rather than performing scRNAseq, we tackled the heterogeneity question using bulk RNAseq and generating riboprobes with no a priori knowledge to perform a battery of in situ hybridizations for transcriptional heterogeneity in vLGN. By generating riboprobes against single molecules, we delineated distinct populations of transcriptomically and spatially distinct cells, an advantage of this approach over scRNAseq. Conservatively, our results demonstrate the existence of at least a half dozen discrete and separable subtypes of GABAergic neurons in vLGN, (although the number is likely much higher than this) and four distinct, adjacent sublaminae of GABAergic neurons in vLGNe.

Whether there are six GABAergic cell types in vLGN or many more has made us ponder the old question (Cajal, 1893) of *how does one define a cell type?* Traditionally, classes and types of neurons have been characterized on the basis of morphological, electrophysiological, neurochemical, connectomic, or genetic information (Sanes & Masland, 2015). Unfortunately, rarely are all these aspects of neuronal identity accounted for in a comprehensive way to glean a more accurate understanding about the structure of the nervous system. This has led to discrepant subtype classification across technical methodologies and challenges to comparing results between research groups. Here, we used spatial distribution and transcriptional profiles to classify neurons into distinct subtypes. Molecular markers remain at present a leading characteristic for such classifications, though they are not without their limitations. It may well be that vLGN neurons can be further subdivided if classified by 2–3 molecular markers rather than one (as we observed in *Spp1* ^+^ and *Pvalb* ^+^ neurons). However, the very fact that differential expression of one molecule was sufficient to differentiate two vLGN populations is a strong indicator that the nucleus as a whole is more diversely populated than previously appreciated. Nevertheless, we acknowledge here the efforts to create a more comprehensive framework to classifying cell types in the field. A set of recent studies represent a major step towards this goal by utilizing Patch‐seq to simultaneously characterize cortical GABAergic neurons electrophysiologically, morphologically, and transcriptomically (Gouwens, Sorensen, & Baftizadeh, 2020; Scala et al., 2020). Our approach here did not take into account these additional functional aspects of neuronal identity for all of the GABAergic cell types identified.

Our data, when taken together, suggest the possibility that functional organization of non‐image‐forming information from retina to vLGN is extracted from the segregation of transcriptionally distinct retinorecipient cells. We view these results as a framework for further dissecting the structure, circuitry, and functions of the vLGN at a cell type‐specific level. How this heterogeneity and organization contributes to the yet‐to‐be determined functions of the vLGN remains to be defined.

## CONFLICTS OF INTEREST DISCLOSURE

The authors have no conflicts of interest to declare.

### OPEN RESEARCH BADGES

This article has received a badge for *Open Materials* because it provided all relevant information to reproduce the study in the manuscript. More information about the Open Science badges can be found at https://cos.io/our‐services/open‐science‐badges/.

## Supporting information

Supplementary Material

## References

[jnc15101-bib-0001] Arcelli, P. , Frassoni, C. , Regondi, M. , Biasi, S. , & Spreafico, R. (1997). GABAergic neurons in mammalian thalamus: A marker of thalamic complexity? Brain Research Bulletin, 42, 27–37. 10.1016/S0361-9230(96)00107-4 8978932

[jnc15101-bib-0002] Berson, D. (2008). Retinal ganglion cell types and their central projections. The Senses: A Comprehensive Reference, 1, 491–520.

[jnc15101-bib-0003] Cadusseau, J. , & Roger, M. (1991). Cortical and subcortical connections of the pars compacta of the anterior pretectal nucleus in the rat. Neuroscience Research, 12, 83–100. 10.1016/0168-0102(91)90102-5 1721119

[jnc15101-bib-0004] Cajal, S. R. (1893). La retine des vertebres. Cellule, 9, 119–255.

[jnc15101-bib-0005] Charalambakis, N. E. , Govindaiah, G. , Campbell, P. W. , & Guido, W. (2019). Developmental remodeling of thalamic interneurons requires retinal signaling. Journal of Neuroscience, 39, 3856–3866. 10.1523/JNEUROSCI.2224-18.2019 30842249 PMC6520504

[jnc15101-bib-0006] Cruz‐Martín, A. , El‐Danaf, R. N. , Osakada, F. , Sriram, B. , Dhande, O. S. , Nguyen, P. L. , … Huberman, A. D. (2014). A dedicated circuit links direction‐selective retinal ganglion cells to the primary visual cortex. Nature, 507, 358–361. 10.1038/nature12989 24572358 PMC4143386

[jnc15101-bib-0007] Dhande, O. S. , Hua, E. W. , Guh, E. , Yeh, J. , Bhatt, S. , Zhang, Y. , … Crair, M. C. (2011). Development of single retinofugal axon arbors in normal and β2 knock‐out mice. The Journal of Neuroscience, 31, 3384–3399. 21368050 10.1523/JNEUROSCI.4899-10.2011 PMC3060716

[jnc15101-bib-0008] Dhande, O. S. , & Huberman, A. D. (2014). Retinal ganglion cell maps in the brain: Implications for visual processing. Current Opinion in Neurobiology, 24, 133–142. 10.1016/j.conb.2013.08.006 24492089 PMC4086677

[jnc15101-bib-0009] Dhande, O. S. , Stafford, B. K. , Lim, J.‐H.‐A. , & Huberman, A. D. (2015). Contributions of retinal ganglion cells to subcortical visual processing and behaviors. Annual Review of Vision Science, 1, 291–328. 10.1146/annurev-vision-082114-035502 28532372

[jnc15101-bib-0010] El‐Danaf, R. N. , Krahe, T. E. , Dilger, E. K. , Bickford, M. E. , Fox, M. A. , & Guido, W. (2015). Developmental remodeling of relay cells in the dorsal lateral geniculate nucleus in the absence of retinal input. Neural Development, 10, 19. 10.1186/s13064-015-0046-6 26174426 PMC4502538

[jnc15101-bib-0011] Evangelio, M. , García‐Amado, M. , & Clascá, F. (2018). Thalamocortical projection neuron and interneuron numbers in the visual thalamic nuclei of the adult C57BL/6 mouse. Frontiers in Neuroanatomy, 12, 27. 10.3389/fnana.2018.00027 29706872 PMC5906714

[jnc15101-bib-0012] Fleming, M. D. , Benca, R. M. , & Behan, M. (2006). Retinal projections to the subcortical visual system in congenic albino and pigmented rats. Neuroscience, 143, 895–904. 10.1016/j.neuroscience.2006.08.016 16996223 PMC1876705

[jnc15101-bib-0013] Gabbott, P. , & Bacon, S. (1994). An oriented framework of neuronal processes in the ventral lateral geniculate nucleus of the rat demonstrated by NADPH diaphorase histochemistry and GABA immunocytochemistry. Neuroscience, 60, 417–440. 10.1016/0306-4522(94)90254-2 7521023

[jnc15101-bib-0014] Gaillard, F. , Karten, H. J. , & Sauvé, Y. (2013). Retinorecipient areas in the diurnal murine rodent Arvicanthis niloticus: A disproportionally large superior colliculus. Journal of Comparative Neurology, 521, 1699–1726. 10.1002/cne.23303 23322547

[jnc15101-bib-0015] Gale, S. D. , & Murphy, G. J. (2014). Distinct representation and distribution of visual information by specific cell types in mouse superficial superior colliculus. Journal of Neuroscience, 34, 13458–13471. 10.1523/JNEUROSCI.2768-14.2014 25274823 PMC4180477

[jnc15101-bib-0016] Gale, S. D. , & Murphy, G. J. (2016). Active dendritic properties and local inhibitory input enable selectivity for object motion in mouse superior colliculus neurons. Journal of Neuroscience, 36, 9111–9123. 10.1523/JNEUROSCI.0645-16.2016 27581453 PMC6601912

[jnc15101-bib-0017] Godement, P. , Salaün, J. , & Imbert, M. (1984). Prenatal and postnatal development of retinogeniculate and retinocollicular projections in the mouse. Journal of Comparative Neurology, 230, 552–575. 10.1002/cne.902300406 6520251

[jnc15101-bib-0018] Gouwens, N. W. , Sorensen, S. A. , Baftizadeh, F. (2020). Toward an integrated classification of neuronal cell types: Morphoelectric and transcriptomic characterization of individual GABAergic cortical neurons. BioRxiv. 10.1101/2020.02.03.932244

[jnc15101-bib-0019] Gouwens, N. W. , Sorensen, S. A. , Berg, J. , Lee, C. , Jarsky, T. , Ting, J. , … Koch, C. (2019). Classification of electrophysiological and morphological neuron types in the mouse visual cortex. Nature Neuroscience, 22, 1182–1195. 10.1038/s41593-019-0417-0 31209381 PMC8078853

[jnc15101-bib-0020] Guillery, R. (1966). A study of Golgi preparations from the dorsal lateral geniculate nucleus of the adult cat. Journal of Comparative Neurology, 128, 21–49. 10.1002/cne.901280104 4165857

[jnc15101-bib-0021] Hammer, S. , Carrillo, G. L. , Govindaiah, G. , Monavarfeshani, A. , Bircher, J. S. , Su, J. , … Fox, M. A. (2014). Nuclei‐specific differences in nerve terminal distribution, morphology, and development in mouse visual thalamus. Neural Development, 9, 1. 10.1186/1749-8104-9-16 25011644 PMC4108237

[jnc15101-bib-0022] Harrington, M. E. (1997). The ventral lateral geniculate nucleus and the intergeniculate leaflet: Interrelated structures in the visual and circadian systems. Neuroscience & Biobehavioral Reviews, 21, 705–727. 10.1016/S0149-7634(96)00019-X 9353800

[jnc15101-bib-0023] Hattar, S. , Kumar, M. , Park, A. , Tong, P. , Tung, J. , Yau, K. W. , & Berson, D. M. (2006). Central projections of melanopsin‐expressing retinal ganglion cells in the mouse. Journal of Comparative Neurology, 497, 326–349. 10.1002/cne.20970 PMC2885916 16736474

[jnc15101-bib-0024] He, J. , Xu, X. , Monavarfeshani, A. , Banerjee, S. , Fox, M. A. , & Xie, H. (2019). Retinal‐input‐induced epigenetic dynamics in the developing mouse dorsal lateral geniculate nucleus. Epigenetics & Chromatin, 12, 13. 10.1186/s13072-019-0257-x 30764861 PMC6374911

[jnc15101-bib-0025] Hickey, T. , & Spear, P. (1976). Retinogeniculate projections in hooded and albino rats: An autoradiographic study. Experimental Brain Research, 24, 523–529. 10.1007/BF00234968 1253865

[jnc15101-bib-0026] Hong, Y. K. , & Chen, C. (2011). Wiring and rewiring of the retinogeniculate synapse. Current Opinion in Neurobiology, 21, 228–237. 10.1016/j.conb.2011.02.007 21558027 PMC3099477

[jnc15101-bib-0027] Hoy, J. L. , Bishop, H. I. , & Niell, C. M. (2019). Defined cell types in superior colliculus make distinct contributions to prey capture behavior in the mouse. Current Biology, 29, 4130–4138, e4135. 10.1016/j.cub.2019.10.017 31761701 PMC6925587

[jnc15101-bib-0028] Huang, L. U. , Xi, Y. , Peng, Y. , Yang, Y. , Huang, X. , Fu, Y. , … Ren, C. (2019). A visual circuit related to habenula underlies the antidepressive effects of light therapy. Neuron, 102, 128 – 142, e128. 10.1016/j.neuron.2019.01.037 30795900

[jnc15101-bib-0029] Huberman, A. D. , Manu, M. , Koch, S. M. , Susman, M. W. , Lutz, A. B. , Ullian, E. M. , … Barres, B. A. (2008). Architecture and activity‐mediated refinement of axonal projections from a mosaic of genetically identified retinal ganglion cells. Neuron, 59, 425–438. 10.1016/j.neuron.2008.07.018 18701068 PMC8532044

[jnc15101-bib-0030] Huberman, A. D. , Wei, W. , Elstrott, J. , Stafford, B. K. , Feller, M. B. , & Barres, B. A. (2009). Genetic identification of an On‐Off direction‐selective retinal ganglion cell subtype reveals a layer‐specific subcortical map of posterior motion. Neuron, 62, 327–334. 19447089 10.1016/j.neuron.2009.04.014 PMC3140054

[jnc15101-bib-0031] Inamura, N. , Ono, K. , Takebayashi, H. , Zalc, B. , & Ikenaka, K. (2011). Olig2 lineage cells generate GABAergic neurons in the prethalamic nuclei, including the zona incerta, ventral lateral geniculate nucleus and reticular thalamic nucleus. Developmental Neuroscience, 33, 118–129. 10.1159/000328974 21865661

[jnc15101-bib-0032] Jager, P. , Ye, Z. , Yu, X. , Zagoraiou, L. , Prekop, H.‐T. , Partanen, J. … Delogu, A. (2016). Tectal‐derived interneurons contribute to phasic and tonic inhibition in the visual thalamus. Nature Communications, 7(1), 1–14. 10.1038/ncomms13579 PMC5155147 27929058

[jnc15101-bib-0033] Jaubert‐Miazza, L. , Green, E. , Lo, F.‐S. , Bui, K. , Mills, J. , & Guido, W. (2005). Structural and functional composition of the developing retinogeniculate pathway in the mouse. Visual Neuroscience, 22, 661–676. 16332277 10.1017/S0952523805225154

[jnc15101-bib-0034] Kalish, B. T. , Cheadle, L. , Hrvatin, S. , Nagy, M. A. , Rivera, S. , Crow, M. , … Greenberg, M. E. (2018). Single‐cell transcriptomics of the developing lateral geniculate nucleus reveals insights into circuit assembly and refinement. Proceedings of the National Academy of Sciences, 115(5), E1051–E1060. 10.1073/pnas.1717871115 PMC5798372 29343640

[jnc15101-bib-0035] Kay, J. N. , De la Huerta, I. , Kim, I.‐J. , Zhang, Y. , Yamagata, M. , Chu, M. W. , … Sanes, J. R. (2011). Retinal ganglion cells with distinct directional preferences differ in molecular identity, structure, and central projections. The Journal of Neuroscience, 31, 7753–7762. 10.1523/JNEUROSCI.0907-11.2011 21613488 PMC3108146

[jnc15101-bib-0036] Kerschensteiner, D. , & Guido, W. (2017). Organization of the dorsal lateral geniculate nucleus in the mouse. Visual Neuroscience, 34, E008–E008. 10.1017/S0952523817000062 28965501 PMC6380502

[jnc15101-bib-0037] Kim, I.‐J. , Zhang, Y. , Meister, M. , & Sanes, J. R. (2010). Laminar restriction of retinal ganglion cell dendrites and axons: Subtype‐specific developmental patterns revealed with transgenic markers. The Journal of Neuroscience, 30, 1452–1462. 10.1523/JNEUROSCI.4779-09.2010 20107072 PMC2822471

[jnc15101-bib-0038] Kim, I.‐J. , Zhang, Y. , Yamagata, M. , Meister, M. , & Sanes, J. R. (2008). Molecular identification of a retinal cell type that responds to upward motion. Nature, 452, 478–482. 10.1038/nature06739 18368118

[jnc15101-bib-0039] Krahe, T. E. , El‐Danaf, R. N. , Dilger, E. K. , Henderson, S. C. , & Guido, W. (2011). Morphologically distinct classes of relay cells exhibit regional preferences in the dorsal lateral geniculate nucleus of the mouse. The Journal of Neuroscience, 31, 17437–17448. 10.1523/JNEUROSCI.4370-11.2011 22131405 PMC6623799

[jnc15101-bib-0040] Krienen, F. M. , Goldman, M. , Zhang, Q. (2019). Innovations in Primate InterneuronRepertoire., bioRxiv, 709501. 10.1101/709501

[jnc15101-bib-0041] Langel, J. , Ikeno, T. , Yan, L. , Nunez, A. A. , & Smale, L. (2018). Distributions of GABAergic and glutamatergic neurons in the brains of a diurnal and nocturnal rodent. Brain Research, 1700, 152–159. 10.1016/j.brainres.2018.08.019 30153458

[jnc15101-bib-0042] Leist, M. , Datunashvilli, M. , Kanyshkova, T. , Zobeiri, M. , Aissaoui, A. , Cerina, M. , … Budde, T. (2016). Two types of interneurons in the mouse lateral geniculate nucleus are characterized by different h‐current density. Scientific Reports, 6, 24904. 10.1038/srep24904 27121468 PMC4848471

[jnc15101-bib-0043] Lim, L. , Mi, D. , Llorca, A. , & Marín, O. (2018). Development and functional diversification of cortical interneurons. Neuron, 100, 294–313. 10.1016/j.neuron.2018.10.009 30359598 PMC6290988

[jnc15101-bib-0044] Ling, C. , Hendrickson, M. L. , & Kalil, R. E. (2012). Morphology, classification, and distribution of the projection neurons in the dorsal lateral geniculate nucleus of the rat. PLoS One, 7, e49161. 10.1371/journal.pone.0049161 23139837 PMC3489731

[jnc15101-bib-0045] Martersteck, E. M. , Hirokawa, K. E. , Evarts, M. , Bernard, A. , Duan, X. , Li, Y. , … Harris, J. A. (2017). Diverse central projection patterns of retinal ganglion cells. Cell Reports, 18, 2058–2072. 10.1016/j.celrep.2017.01.075 28228269 PMC5357325

[jnc15101-bib-0046] Martin, P. (1986). The projection of different retinal ganglion cell classes to the dorsal lateral geniculate nucleus in the hooded rat. Experimental Brain Research, 62, 77–88. 10.1007/BF00237404 3956639

[jnc15101-bib-0047] Mo, A. , Mukamel, E. A. , Davis, F. P. , Luo, C. , Henry, G. L. , Picard, S. , … Nathans, J. (2015). Epigenomic signatures of neuronal diversity in the mammalian brain. Neuron, 86, 1369–1384. 10.1016/j.neuron.2015.05.018 26087164 PMC4499463

[jnc15101-bib-0048] Monavarfeshani, A. , Sabbagh, U. , & Fox, M. A. (2017). Not a one‐trick pony: Diverse connectivity and functions of the rodent lateral geniculate complex. Visual Neuroscience, 34. 10.1017/S0952523817000098 PMC5755970 28965517

[jnc15101-bib-0049] Monavarfeshani, A. , Stanton, G. , Van Name, J. , Su, K. , Mills, W. A. , Swilling, K. , … Fox, M. A. (2018). ). LRRTM1 underlies synaptic convergence in visual thalamus. eLife, 7, e33498. 10.7554/eLife.33498 29424692 PMC5826289

[jnc15101-bib-0050] Morin, L. P. , & Studholme, K. M. (2014). Retinofugal projections in the mouse. Journal of Comparative Neurology, 522, 3733–3753. 10.1002/cne.23635 PMC4142087 24889098

[jnc15101-bib-0051] Muscat, L. , Huberman, A. D. , Jordan, C. L. , & Morin, L. P. (2003). Crossed and uncrossed retinal projections to the hamster circadian system. Journal of Comparative Neurology, 466, 513–524. 10.1002/cne.10894 14566946

[jnc15101-bib-0052] Niimi, K. , Kanaseki, T. , & Takimoto, T. (1963). The comparative anatomy of the ventral nucleus of the lateral geniculate body in mammals. Journal of Comparative Neurology, 121, 313–323. 10.1002/cne.901210303 14100018

[jnc15101-bib-0053] Oh, S. W. , Harris, J. A. , Ng, L. , Winslow, B. , Cain, N. , Mihalas, S. , … Zeng, H. (2014). A mesoscale connectome of the mouse brain. Nature, 508, 207–214. 10.1038/nature13186 24695228 PMC5102064

[jnc15101-bib-0054] Oliveira, A. F. , & Yonehara, K. (2018). The mouse superior colliculus as a model system for investigating cell type‐based mechanisms of visual motor transformation. Frontiers in Neural Circuits, 12, 59. 10.3389/fncir.2018.00059 30140205 PMC6094993

[jnc15101-bib-0055] Osterhout, J. A. , Josten, N. , Yamada, J. , Pan, F. , Wu, S.‐W. , Nguyen, P. L. , … Huberman, A. D. (2011). Cadherin‐6 mediates axon‐target matching in a non‐image‐forming visual circuit. Neuron, 71, 632–639. 10.1016/j.neuron.2011.07.006 21867880 PMC3513360

[jnc15101-bib-0056] Peng, Y.‐R. , Shekhar, K. , Yan, W. , Herrmann, D. , Sappington, A. , Bryman, G. S. , … Sanes, J. R. (2019). Molecular classification and comparative taxonomics of foveal and peripheral cells in primate retina. Cell, 176(1222–1237), e1222. 10.1016/j.cell.2019.01.004 PMC6424338 30712875

[jnc15101-bib-0057] Petrof, I. , & Sherman, S. M. (2013). Functional significance of synaptic terminal size in glutamatergic sensory pathways in thalamus and cortex. The Journal of Physiology, 591, 3125–3131. 10.1113/jphysiol.2012.247619 23359668 PMC3717215

[jnc15101-bib-0058] Piscopo, D. M. , El‐Danaf, R. N. , Huberman, A. D. , & Niell, C. M. (2013). Diverse visual features encoded in mouse lateral geniculate nucleus. The Journal of Neuroscience, 33, 4642–4656. 10.1523/JNEUROSCI.5187-12.2013 23486939 PMC3665609

[jnc15101-bib-0059] Reese, B. (1988). ‘Hidden lamination’in the dorsal lateral geniculate nucleus: The functional organization of this thalamic region in the rat. Brain Research Reviews, 13, 119–137. 10.1016/0165-0173(88)90017-3 3289687

[jnc15101-bib-0060] Reinhard, K. , Li, C. , Do, Q. , Burke, E. G. , Heynderickx, S. , & Farrow, K. (2019). A projection specific logic to sampling visual inputs in mouse superior colliculus. Elife, 8. 10.7554/eLife.50697 PMC6872211 31750831

[jnc15101-bib-0061] Ribak, C. E. , & Peters, A. (1975). An autoradiographic study of the projections from the lateral geniculate body of the rat. Brain Research, 92, 341–368. 10.1016/0006-8993(75)90322-4 1174957

[jnc15101-bib-0062] Rivlin‐Etzion, M. , Zhou, K. , Wei, W. , Elstrott, J. , Nguyen, P. L. , Barres, B. A. , … Feller, M. B. (2011). Transgenic mice reveal unexpected diversity of on‐off direction‐selective retinal ganglion cell subtypes and brain structures involved in motion processing. The Journal of Neuroscience, 31, 8760–8769. 10.1523/JNEUROSCI.0564-11.2011 21677160 PMC3139540

[jnc15101-bib-0063] Sabbagh, U. , Govindaiah, G. , Somaiya, R. D. , Ha, R. V. , Wei, J. C. , Guido, W. , & Fox, M. A. (2020) Diverse GABAergic neurons organize into subtype‐specific sublaminae in the ventral lateral geniculate nucleus. Journal of Neurochemistry. bioRxiv, 2020.2005.2003.073197. 10.1101/2020.05.03.073197 PMC8210463 32497303

[jnc15101-bib-0064] Sabbagh, U. , Monavarfeshani, A. , Su, K. , Zabet‐Moghadam, M. , Cole, J. , Carnival, E. , … Fox, M. A. (2018). Distribution and development of molecularly distinct perineuronal nets in visual thalamus. Journal of Neurochemistry, 147(5), 626–646. 10.1111/jnc.14614 30326149 PMC6532419

[jnc15101-bib-0065] Sanes, J. R. , & Masland, R. H. (2015). The types of retinal ganglion cells: Current status and implications for neuronal classification. Annual Review of Neuroscience, 38, 221–246. 10.1146/annurev-neuro-071714-034120 25897874

[jnc15101-bib-0066] Saunders, A. , Macosko, E. Z. , Wysoker, A. , Goldman, M. , Krienen, F. M. , de Rivera, H. , … McCarroll, S. A. (2018). Molecular diversity and specializations among the cells of the adult mouse brain. Cell, 174(1015–1030), e1016. 10.1016/j.cell.2018.07.028 PMC6447408 30096299

[jnc15101-bib-0067] Scala, F. , Kobak, D. , Bernabucci, M. , (2020) Phenotypic variation within and across transcriptomic cell types in mouse motor cortex. bioRxiv. 10.1101/2020.02.03.929158 PMC8113357 33184512

[jnc15101-bib-0068] Seabrook, T. , Burbridge, T. , Crair, M. , & Huberman, A. (2017). Architecture, function, and assembly of the mouse visual system. Annual Review of Neuroscience, 40, 499. 10.1146/annurev-neuro-071714-033842 28772103

[jnc15101-bib-0069] Seabrook, T. A. , Krahe, T. E. , Govindaiah, G. , & Guido, W. (2013). Interneurons in the mouse visual thalamus maintain a high degree of retinal convergence throughout postnatal development. Neural Development, 8, 1. 10.1186/1749-8104-8-24 24359973 PMC3878090

[jnc15101-bib-0070] Shang, C. , Chen, Z. , Liu, A. , Li, Y. , Zhang, J. , Qu, B. , … Cao, P. (2018). Divergent midbrain circuits orchestrate escape and freezing responses to looming stimuli in mice. Nature Communications, 9, 1–17. 10.1038/s41467-018-03580-7 PMC5964329 29581428

[jnc15101-bib-0071] Shang, C. , Liu, Z. , Chen, Z. , Shi, Y. , Wang, Q. , Liu, S. , … Cao, P. (2015). A parvalbumin‐positive excitatory visual pathway to trigger fear responses in mice. Science, 348, 1472–1477. 10.1126/science.aaa8694 26113723

[jnc15101-bib-0072] Su, J. , Charalambakis, N. E. , Sabbagh, U. , Somaiya, R. D. , Monavarfeshani, A. , Guido, W. , & Fox, M. A. (2020). Retinal inputs signal astrocytes to recruit interneurons into visual thalamus. Proceedings of the National Academy of Sciences, 117, 2671–2682. 10.1073/pnas.1913053117 PMC7007527 31964831

[jnc15101-bib-0073] Su, J. , Gorse, K. , Ramirez, F. , & Fox, M. A. (2010). Collagen XIX is expressed by interneurons and contributes to the formation of hippocampal synapses. Journal of Comparative Neurology, 518, 229–253. 10.1002/cne.22228 PMC2913722 19937713

[jnc15101-bib-0074] Su, J. , Haner, C. V. , Imbery, T. E. , Brooks, J. M. , Morhardt, D. R. , Gorse, K. , … Fox, M. A. (2011). Reelin is required for class‐specific retinogeniculate targeting. The Journal of Neuroscience, 31, 575–586. 10.1523/JNEUROSCI.4227-10.2011 21228166 PMC3257181

[jnc15101-bib-0075] Swanson, L. , Cowan, W. , & Jones, E. (1974). An autoradiographic study of the efferent connections of the ventral lateral geniculate nucleus in the albino rat and the cat. Journal of Comparative Neurology, 156, 143–163. 10.1002/cne.901560203 4425296

[jnc15101-bib-0076] Trejo, L. J. , & Cicerone, C. M. (1984). Cells in the pretectal olivary nucleus are in the pathway for the direct light reflex of the pupil in the rat. Brain Research, 300, 49–62. 10.1016/0006-8993(84)91340-4 6733467

[jnc15101-bib-0077] Tremblay, R. , Lee, S. , & Rudy, B. (2016). GABAergic interneurons in the neocortex: From cellular properties to circuits. Neuron, 91, 260–292. 10.1016/j.neuron.2016.06.033 27477017 PMC4980915

[jnc15101-bib-0078] Wang, Q. , Marcucci, F. , Cerullo, I. , & Mason, C. (2016). Ipsilateral and contralateral retinal ganglion cells express distinct genes during decussation at the optic chiasm. Eneuro, 3(6). 10.1523/ENEURO.0169-16.2016 PMC5136615 27957530

[jnc15101-bib-0079] Yonehara, K. , Ishikane, H. , Sakuta, H. , Shintani, T. , Nakamura‐Yonehara, K. , Kamiji, N. L. , … Noda, M. (2009). Identification of retinal ganglion cells and their projections involved in central transmission of information about upward and downward image motion. PLoS One, 4(1), e4320. 10.1371/journal.pone.0004320 19177171 PMC2629575

[jnc15101-bib-0080] Yuge, K. , Kataoka, A. , Yoshida, A. C. , Itoh, D. , Aggarwal, M. , Mori, S. , … Shimogori, T. (2011). Region‐specific gene expression in early postnatal mouse thalamus. Journal of Comparative Neurology, 519, 544–561. 10.1002/cne.22532 21192083

[jnc15101-bib-0081] Zingg, B. , Chou, X.‐L. , Zhang, Z.‐G. , Mesik, L. , Liang, F. , Tao, H. W. , & Zhang, L. I. (2017). AAV‐mediated anterograde transsynaptic tagging: Mapping corticocollicular input‐defined neural pathways for defense behaviors. Neuron, 93, 33–47. 10.1016/j.neuron.2016.11.045 27989459 PMC5538794

