# Supplementary material for: Diverse GABAergic neurons organize into subtype‐specific sublaminae in the ventral lateral geniculate nucleus
Source: J Neurochem. 2020 Jun 24;159(3):479–97. doi: 10.1111/jnc.15101 (PMC8210463; doi:10.1111/jnc.15101)
Supplement: Supplementary file 1 — Supplementary Material [file JNC-159-479-s001.zip › jnc15101-sup-0001-Supinfo.docx]

**Diverse GABAergic neurons organize into subtype-specific sublaminae in the ventral lateral geniculate nucleus**

**Ubadah Sabbagh^1,2^, Gubbi Govindaiah^3^, Rachana D. Somaiya^1,2^, Ryan V. Ha^4^, Jessica C. Wei^5^, William Guido^3^, Michael A. Fox^1,4,6,7,#^**

^1^Center for Neurobiology Research, Fralin Biomedical Research Institute at Virginia Tech Carilion, Roanoke, VA

^2^Graduate Program in Translational Biology, Medicine, and Health, Virginia Tech, Blacksburg, VA

^3^Department of Anatomical Sciences and Neurobiology, University of Louisville School of Medicine, Louisville, KY

^4^School of Neuroscience, Virginia Tech, Blacksburg, VA

^5^NeuroSURF, Fralin Biomedical Research Institute at Virginia Tech Carilion, Roanoke, VA

^6^Department of Biological Sciences, Virginia Tech, Blacksburg, VA

^7^Department of Pediatrics, Virginia Tech Carilion School of Medicine, Roanoke, VA

^#^ **Corresponding author: Michael A. Fox**

Director, Center for Neurobiology Research

Fralin Biomedical Research Institute at Virginia Tech Carilion

2 Riverside Circle, Roanoke, VA 24016

[mafox1@vtc.vt.edu](mailto:mafox1@vtc.vt.edu)

**This pdf contains 1 table (Supplemental Table S1).**

|  | **Gene** | **SCN** | **dLGN** | **vLGN** | **SC** |  |  | **Gene** | **SCN** | **dLGN** | **vLGN** | **SC** |
| --- | --- | --- | --- | --- | --- | --- | --- | --- | --- | --- | --- | --- |
| 1 | *Adcyap1r1* | + | + | +++ | + |  | 36 | *Sh3bgrl2* | – | + | + | + |
| 2 | *Ankrd34b* | – | – | ++ | - |  | 37 | *Slc39a14* | – | – | ++ | + |
| 3 | *Anxa3* | – | – | + | – |  | 38 | *Spp1* | – | – | ++ | + |
| 4 | *Arx* | – | – | ++ | – |  | 39 | *Sst* | n.d. | n.d. | ++ | ++ |
| 5 | *Asic4* | – | + | +++ | +++ |  | 40 | *Steap2* | – | – | + | – |
| 6 | *Atpaf1* | – | ++ | + | + |  | 41 | *Tcf7l2* | – | +++ | + | + |
| 7 | *Cabp7* | – | – | ++ | + |  | 42 | *Unc5d* | – | – | + | – |
| 8 | *Calb1* | + | – | ++ | ++ |  | 43 | *Zfp804a* | – | +++ | + | – |
| 9 | *Cbln4* | – | – | + | + |  |  |  |  |  |  |  |
| 10 | *Cd24a* | +++ | – | + | + |  |  |  |  |  |  |  |
| 11 | *Chrm2* | – | + | ++ | ++ |  |  |  |  |  |  |  |
| 12 | *Chst2* | – | + | + | ++ |  |  |  |  |  |  |  |
| 13 | *Cntn4* | – | + | + | – |  |  |  |  |  |  |  |
| 14 | *Col15a1* | + | – | + | – |  |  |  |  |  |  |  |
| 15 | *Coro6* | – | +++ | + | + |  |  |  |  |  |  |  |
| 16 | *Ecel1* | – | – | ++ | + |  |  |  |  |  |  |  |
| 17 | *Gad1* | +++ | + | +++ | +++ |  |  |  |  |  |  |  |
| 18 | *Gad2* | +++ | + | +++ | +++ |  |  |  |  |  |  |  |
| 19 | *Grik1* | ++ | ++ | + | + |  |  |  |  |  |  |  |
| 20 | *Islr2* | + | – | +++ | n.d. |  |  |  |  |  |  |  |
| 21 | *Lmo3* | – | – | + | + |  |  |  |  |  |  |  |
| 22 | *Loc433436* | – | – | + | – |  |  |  |  |  |  |  |
| 23 | *Lypd1* | – | – | ++ | – |  |  |  |  |  |  |  |
| 24 | *Nacc2* | – | + | + | + |  |  |  |  |  |  |  |
| 25 | *Nos1* | – | – | + | – |  |  |  |  |  |  |  |
| 26 | *Nos1ap* | ++ | + | + | + |  |  |  |  |  |  |  |
| 27 | *Nxph1* | – | – | ++ | – |  |  |  |  |  |  |  |
| 28 | *Pvalb* | – | – | + | + |  |  |  |  |  |  |  |
| 29 | *Pcdh11x* | + | – | + | + |  |  |  |  |  |  |  |
| 30 | *Penk* | ++ | – | ++ | + |  |  |  |  |  |  |  |
| 31 | *Prkcd* | – | +++ | + | – |  |  |  |  |  |  |  |
| 32 | *Ptprk* | – | – | + | – |  |  |  |  |  |  |  |
| 33 | *Rab37* | – | +++ | + | – |  |  |  |  |  |  |  |
| 34 | *Sdk1* | n.d. | – | + | n.d. |  |  |  |  |  |  |  |
| 35 | *Sdk2* | n.d. | – | + | n.d. |  |  |  |  |  |  |  |

**Table S1**. **Riboprobe screen of genes enriched in vLGN.** Symbols are qualitative indicators of expression in each region. The minus symbol indicates no cells expressing this mRNA observed and plus symbols indicate that cells were observed, ranging from some (+) to many (+++). SCN – suprachiasmatic nucleus; dLGN – dorsal lateral geniculate nucleus; vLGN – ventral lateral geniculate nucleus; SC – superior colliculus; n.d. – not done.
